# Multi-omics analysis of LAMB3 as a potential immunological and biomarker in pan-cancer

**DOI:** 10.3389/fmolb.2023.1157970

**Published:** 2023-07-27

**Authors:** Yanghao Wang, Guoyu Li, ZhiYuan Wang, Weizhou Wang, Xiaofang Wang, Xuan Luo, Juan Zhao, Fangfang Li, Li Bian

**Affiliations:** ^1^ Department of Pathology, The First Affiliated Hospital of Kunming Medical University, Kunming, Yunnan, China; ^2^ Department of Orthopedics, The First Affiliated Hospital of Kunming Medical University, Kunming, Yunnan, China; ^3^ Department of Pathology, The Second Affiliated Hospital of Kunming Medical University, Kunming, Yunnan, China

**Keywords:** LAMB3, tumor immunity, multi-omics, pan-cancer, biomarker

## Abstract

Laminin Subunit Beta 3 (LAMB3) is a transcription factor and participates in the coding of laminin. It plays an important role in cell proliferation, adhesion, and transfer by regulating various target genes and signaling pathways. However, the role of LAMB3 in human pan-cancer immunology and prognosis is still poorly understood. The TCGA, GTEx, CCLE, and HPA databases were utilized for the analysis of LAMB mRNA and protein expression. The expression of LAMB3 in various immune and molecular subtypes of human cancer was examined using the TISIDB database. The prognostic significance of LAMB3 in various cancers and clinical subtypes was investigated using Kaplan-Meier and Cox regression analysis. The relationship between LAMB3 expression, various immune cell infiltration, immune checkpoints, tumor mutational load, microsatellite instability, and DNA methylation was examined using the TCGA database. Clinical samples of four lung cancer cell lines and eight lung cancer cases were collected to confirm the expression of mRNA in lung cancer. In 17 cancers, the mRNA for LAMB3 is expressed differently and has good diagnostic and prognostic value in 22 cancers. Cox regression and Nomogram analysis show that LAMB3 is an independent risk factor for 15 cancers. LAMB3 is implicated in a variety of tumorigenesis and immune-related signaling pathways, according to GO, KEGG, and GSEA results. LAMB3 expression was associated with TMB in 33 cancer types and MSI in 32 cancer types, while in lung cancer LAMB3 expression was strongly associated with immune cell infiltration and negatively correlated with all seven methylated CpG islands. Cellular experiments demonstrated that LAMB3 promotes malignant behavior of tumor cells. Preliminary mechanistic exploration revealed its close association with PD-L1, CTLA4, cell stemness gene CD133 and β-catenin-related signaling pathways. Based on these findings, it appears that LAMB3 could be a potential therapeutic target for immunotherapy and tumor prognosis. Our findings reveal an important role for LAMB3 in different cancer types.

## 1 Introduction

The global incidence and mortality of cancer are increasing year by year, seriously affecting the safety of public health and the quality of human life (Chen et al., 2021). Despite the efforts of scientists to cure cancer, there is no absolute cure for cancer (Ribas and Wolchok, 2018). It has become a new research method to find diagnostic biomarkers and therapeutic methods for cancer through large data synthesis analysis with the development and perfection of cancer public databases like the cancer genome map (TCGA) and the Encyclopedia of Cancer Cell Lines (CCLE) database (Fan et al., 2021; Hu et al., 2021). This method has made important contributions to the precise diagnosis and treatment of cancer.

Laminin plays an important role in regulating cell migration and signal transduction and is necessary for the formation and operation of the basement membrane (Miner and Yurchenco, 2004). Tumor invasion and metastasis are significantly associated with the process of extracellular matrix and tumor breakdown of the basement membrane (Zhu et al., 2020). Laminin Subunit Beta 3 (LAMB3) encodes one of laminin’s three subunits (Huang et al., 2019). Studies have shown that LAMB3 has some association with the prognosis of multiple cancers. Zhou found that LAMB3 single nucleotide polymorphisms (SNPs) could lead to cervical cancer (Zhou et al., 2010). Kwon found that LAMB3 may induce the development of gastric cancer because of promoter demethylation (Kwon et al., 2011). Kinoshita found that LAMB3 high expression had a positive effect on the development of head and neck squamous cell carcinoma (HNSC) (Kinoshita et al., 2012). In addition, LAMB3 is involved in the invasion and metastasis of certain types of cancer (Zhang et al., 2019; Sundqvist et al., 2020; Sui et al., 2022).

Be that as it may, most exploration on the job of LAMB3 in growths is restricted to explicit kinds of disease, LAMB3 the job in human pan-cancer prognosis and immunology is rarely systematically analyzed. We used multiple databases, including TCGA, CCLE, genotype tissue expression (GTEx), human protein mapping (HPA), and cBioPortal to analyze LAMB3 expression levels and prognosis in pan-cancer. We also discussed the potential correlation between LAMB3 expression and the immune checkpoint (ICP) gene, immune cell infiltration level, microsatellite instability (MSI), and tumor mutation load (TMB) in 33 cancers. In addition, to investigate the biological function of LAMB3 in tumors, we carried out an enrichment analysis of LAMB3 co-expressed genes. Finally, we explored differences in LAMB3 expression and prognosis in lung cancer to further verify their outcomes in human cancers. Our results show that LAMB3 are prognostic and a risk factor for multiple cancers. LAMB3 plays an important role in tumor immunity by affecting tumor infiltration immune cells, TMB and MSI. This study provides new insights into the role of LAMB3 in anti-cancer immunotherapy.

## 2 Methods and materials

### 2.1 Expression of LAMB3 gene in pan-cancer

RNA sequencing data and clinical follow-up information for 33 tumor types and normal tissues were downloaded from the TCGA database (https://portal.gdc.cancer.gov/) and the GTEx database (https://gtexportal.org/home/). Data on tumor cell lines are downloaded from the CCLE database (https://portals.broadinstitute.org/ccle/). The BioGPS database (http://biogps.org) is used to analyze the expression of LAMB3 in different cancers and paired normal cell lines. Use R software v3.6.3 for statistical analysis and visualization, and ggplot2 package [version 3.3.3] for visualization. The Mann-Whitney U test detects two sets of data and *p* < 0.05 is considered statistically significant.

### 2.2 Expression of LAMB3 protein in pan-cancer

To assess the differences in LAMB3 expression at the protein level, we downloaded immunohistochemical images of LAMB3 protein expression in normal tissues and their corresponding tumor tissues, including THCA, LUADLUSC, HNSC, SKCM, and STAD, from the HPA database (http://www.proteinatlas.org/) with the top 5 protein expressions and differential gene expression.

### 2.3 Molecular and immune subtypes of LAMB3 in pan-cancer

The correlation between the expression of LAMB3 and the molecular or immune subtype of pan-cancer is discussed in the TISIDB database (http://cis.hku.hk/TISIDB/). The database integrates multiple types of data to assess interactions between tumors and the immune system.

### 2.4 Prognosis and diagnostic value of LAMB3 in pan-cancer

The receiver operating catalytic (ROC) curve is used to assess the diagnostic value of LAMB3 in pan-cancer. We only screened for diseases with AUC >0.7. The relationship between LAMB3 expression and cancer prognosis was evaluated using the Kaplan-Meier diagram (OS, DSS, and PFI). In addition, we further studied the relationship between LAMB3 expression and prognosis (OS, DSS, and PFI) in different clinical subgroups of lung cancer. The forest diagram shows the *p*-value, hazard ratio (HR), and 95% confidence interval. Statistical analysis and visualization using R software v3.6.3. The survminer package [version 0.4.9] is used for visualization. The survival package [version 3.2-10] is used for the statistical analysis of survival data. Statistical testing using Cox regression. *p* < 0.05 is considered statistically significant.

### 2.5 Univariate/multifactorial analysis and the construction of a column diagram

Cox regression analysis (univariate and multivariate analysis) was used to examine the prognostic value of LAMB3 in pan-cancer and its clinical subtypes. Column line plots were constructed using the R package “rms.”

### 2.6 Analysis of LAMB3 expression correlation with immune cells, ICP gene, TMB, and MSI

RNAseq data for cancer (level 3) and corresponding clinical information were obtained from the TCGA database. For reliable immune correlation assessment, we used immunedeconv, which is an R package that integrates six state-of-the-art algorithms including TIMER, xCell, MCP-counter, CIBERSORT, EPIC, and quantized. The expression values of SIGLEC15, IDO1, CD274, HAVCR2, PDCD1, CTLA4, LAG3, and PDCD1LG2 were extracted to observe the expression of immune checkpoint-associated genes. These eight genes are the transcripts associated with immune checkpoints. TMB is derived from the article The Immune Landscape of Cancer published by Thorsson et al. (2018); MSI is derived from the Landscape of Microsatellite Instability Across 39 Cancer Types article published by Bonneville et al. (2017). R package: GSVA package [version 1.34.0] (Hänzelmann et al., 2013). Immuno-infiltration algorithm: ssGSEA (built-in algorithm of GSVA package). Correlation algorithms: MCP-counter and TIMER algorithms. *p* < 0.05 was considered statistically significant.

### 2.7 Protein-protein interaction (PPI) network construction

The first 20 proteins that may bind to the LAMB3 were screened from the STRING website. Cytoscape (version 3.9.0) is applied to the visualization of PPI networks.

### 2.8 Gene ontology (GO), Kyoto encyclopedia of genes and genomes (KEGG), and gene concentration enrichment analysis (GSEA)

GO, KEGG, and GSEA are performed using proteins or differential genes interacting with LAMB3 to analyze the biological and molecular functions of LAMB3 in different cancer types. All of the above analyses were performed and visualized using the R package (version 3.6.3). The ggplot2 package and the cluster Profiler package [version 3.14.3] were used for the above analysis. The reference gene set for GSEA analysis is c5. all.v7.5.1. symbols.gmt [Gene ontology], c6. all.v7.2. symbols.gmt [Oncogenic signatures] and c7. all.v7.5.1. symbols.gmt [Immunologic signatures].

### 2.9 Lung cancer cell lines and normal bronchial epithelial cells in culture

Four lung cancer cell lines (two lung squamous carcinoma, two lung adenocarcinoma) A549, H226, H1299, SK-MES-1, and normal bronchial epithelial cells BEAS-2B. The above cells were routinely cultured in DMEM high sugar medium containing 10% FBS at 37°C and 5% CO_2_.

### 2.10 Clinical sample collection for patients with lung cancer

Lung cancer tissues and their paracancerous tissues from eight patients were collected at the First Affiliated Hospital of Kunming Medical University. All patients signed informed consent and this study was approved by the Ethics Committee of the First Affiliated Hospital of Kunming Medical University.

### 2.11 qRT-PCR validation LAMB3 differential expression in lung cancer

RNA was extracted using the TRIzol method of isolation according to the manufacturer’s instructions (Invitrogen), and template cDNA was synthesized using a reverse transcription kit (Vazyme, 7E581J1). Each gene is amplified with 1 μL template cDNA and 0.6 μL primers (20 μ L system). Denaturate the PCR reaction mixture at 95°C for 10 min and amplify the cDNA template as follows: Stage1 (pre-denaturation): 1 cycle, 95°C 15 min; Stage2 (PCR) Reaction): 95°C denaturation 10 s, 60°C annealing extended 32 s, repeating 40 cycles.

### 2.12 Genetic alterations of LAMB3 in lung cancer

Genetic alterations of LAMB3 in lung cancer were obtained using the cBioPortal database (https://www.cbioportal.org/).

### 2.13 Analyses of the DNA methylation status in the LAMB3 CpG islands

The DNA methylation status of LAMB3 gene CpG sites was analyzed in the TCGA dataset for lung cancer disease using the DiseaseMeth database (http://bio-bigdata.hrbmu.edu.cn/diseasemeth/index.html). RNAseq data in level 3 HTSeq-FPKM format and Illumina human methylation 450 methylation data were downloaded from the lung cancer project of the TCGA database to visualize LAMB3 in relation to multiple CpG loci using ggplot2 [version 3.3.3].

### 2.14 Analysis of co-expression gene of LAMB3 in lung cancer

We screened the top 50 co-expressed genes that were positively and negatively associated with LAMB3 expression in lung cancer. The threshold values were |log2 fold-change (FC)|>2.0 and *P* adj<0.05. Use the stat package for visual analysis.

### 2.15 Exploration of LAMB3 differentially expressed genes (DEGs) in lung cancer

We explored DEGs between different LAMB3 expression groups (low expression group: 0%–50%; high expression group: 50%–100%) in lung cancer using the deseq2 package. The ggplot2 package was used for visualization analysis with a threshold of |log2 fold-change (FC)|>1.0 and *P* adj<0.05. Then, we performed statistical analysis with the cluster Profiler package for GO and KEGG enrichment analysis of DEGs. In addition, a PPI network of DEGs with log2 FC > 1.5 or log2 FC < −2 as the threshold is established by using the STRING network, and the hub gene is analyzed by CytoHubba’s MCC algorithm in Cytoscape (version 3.7.2).

### 2.16 Risk factor models to predict survival

To comprehensively assess patient survival, we constructed a nomogram integrating distinct clinicopathological information using the “rms” package. ROC curves were used to assess the accuracy of the signature prediction. 32 hub genes interacted with LAMB3 in lung cancer. The above 32 genes were subsequently subjected to one-way Cox regression analysis to obtain 7 genes associated with prognosis in lung cancer. Then the least absolute shrinkage and selection operator (LASSO) regression analysis was performed. The LASSO regression could improve the accuracy and interpretability of the model, and eventually build a risk factor model to predict survival.

### 2.17 Western blot (WB)

Cells or exosomes were lysed in RIPA buffer containing 1% PMSF, and the protein concentration was determined using the BCA method. Protein was separated by 10% SDS-PAGE, transferred to PVDF membranes, and incubated with primary antibodies and HRP-coupled secondary antibodies. The membranes were subjected to ECL luminescent solution. The grey-scale values were analyzed using ImageJ software.

### 2.18 Cellular *in vitro* experiments

A549 and H1299 were routinely cultured in DMEM high sugar medium containing 10% FBS at 37°C and 5% CO2. Cells at 40%–60% confluence were transfected with inhibitor LAMB3 for 48 h. Transfection efficiency was observed using fluorescence microscopy and WB. The cells (A549, A549--siLAMB3, H1299, H1299-siLAMB3) were incubated in 96-well plates for 0 h, 24 h, 48 h, and 72 h respectively, and performed with Cell Counting Kit-8 (CCK8) assay. Cells (A549, A549--siLAMB3, H1299, H1299-siLAMB3) were inoculated into Culture-Insert with scratches up to 500 μm wide. The length of scratches was observed and recorded under the microscope after culturing for 0, 6, 12, and 24 h. The area of scratched area was quantified using ImageJ software. The matrix gel was diluted at a ratio of 1:8, and 100 μL of the diluted matrix gel was added to the upper chamber of the Transwell plate, and incubated for 30 min at 37°C. Cell suspension of 200 μL (5 × 104 cells) is added to the upper chamber, and 500 μL of complete medium containing 20% FBS was added to the lower chamber. The plates were incubated for 48 h. The cells were gently wiped off the surface of the matrix gel and the transwell was fixed with 4% paraformaldehyde, and stained with 0.1% crystal violet. Six random fields were counted under the light microscope.

## 3 Results

### 3.1 Differential expression of LAMB3 between tumor and normal tissue samples

**FIGURE 1:**
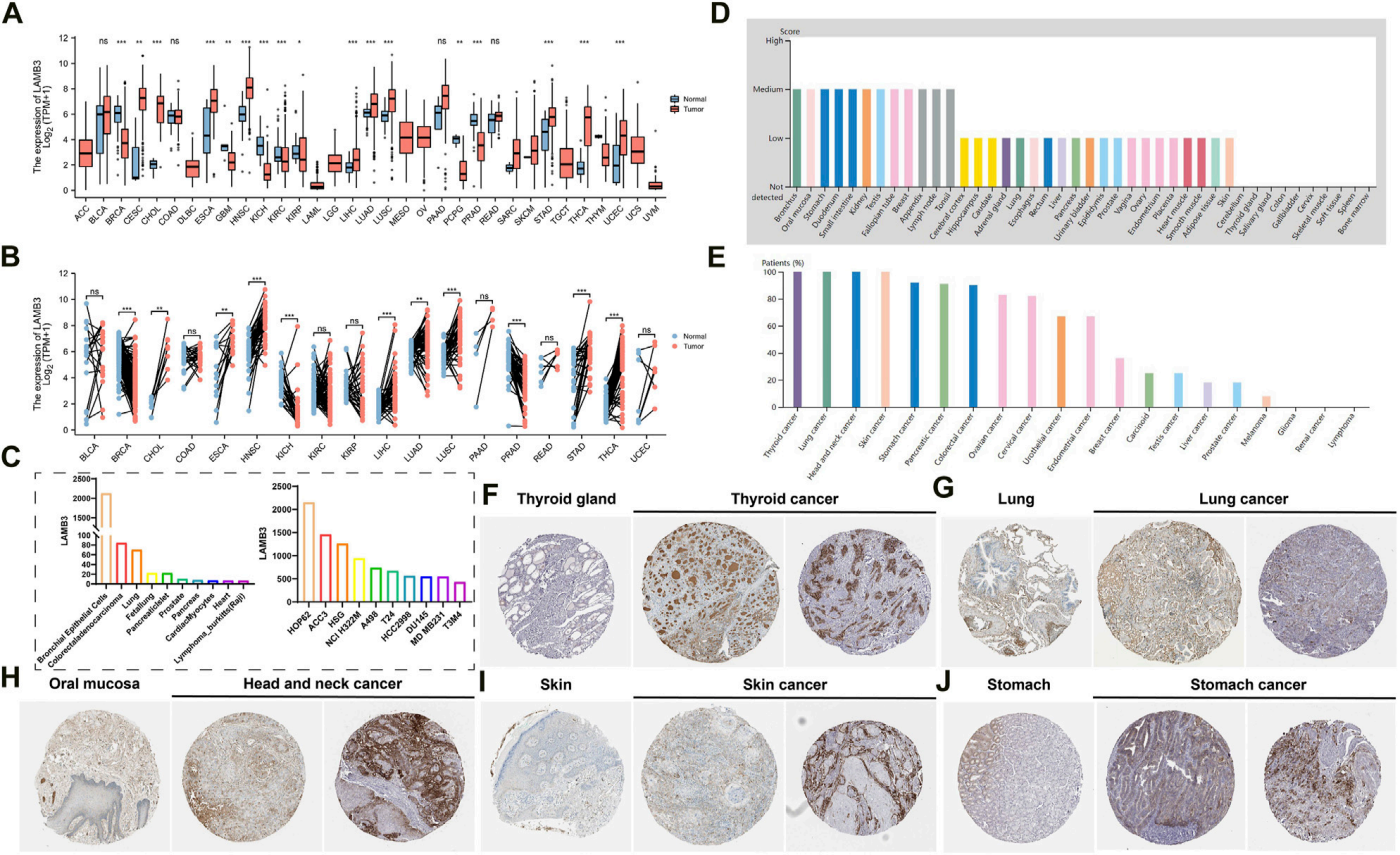
Differential expression of LAMB3. **(A)**. LAMB3 mRNA expression in normal tissues and tumor cell lines (unpaired); **(B)**. LAMB3 mRNA expression in normal tissues and tumor cell lines (paired); **(C)**. LAMB3 mRNA expression in normal tissue cell lines and cancer cell lines; **(D)**. LAMB3 protein expression in normal tissues from the HAP database; **(E)**. LAMB3 protein expression in tumor tissues from the HAP database expression; **(F)**. LAMB3 expression in thyroid and thyroid cancer; **(G)** LAMB3 expression in lung and lung cancer; **(H)**. LAMB3 expression in oral mucosa and head and neck squamous carcinoma; **(I)**. LAMB3 expression in normal skin tissue and skin cancer; **(J)**. LAMB3 expression in stomach and stomach cancer. (*p < 0.05, **p < 0.01, ***p < 0.001).

Combined analysis by the GTEx database and TCGA database showed that LAMB3 mRNA levels were significantly higher in most cancers compared to their normal tissues in unpaired samples, such as CESC, CHOL, ESCA, HNSC, LIHC, LUAD, LUSC, STAD, THCA, UCEC. In contrast, LAMB3 mRNA expression was lower in BRCA, GBM, KICH, KIRC, KIRP, PCPG, and PRAD than in the corresponding normal tissues (Figure 1A). The above trends were the same in the paired samples (Figure 1B). We further investigated the expression of LAMB3 in different cancer cell lines and normal tissues through the BioGPS database, and we found that LAMB3 had high expression in a small number of normal cell strains. But it has high expression levels in almost all cancer lines (Supplementary Table S1). LAMB3 expression levels of the top 10 normal tissue and cancer cell lines are shown in Figure 1C.

In addition, we also analyzed the HPA database’s IHC results and compared them to the gene expression data previously mentioned to assess LAMB3 expression at the protein level. In most cancers, LAMB3 protein expression showed an upward trend over normal tissues (Figures 1D, E). We screened the top 5 cancers (THCA, LUADLUSC, HNSC, SKCM, STAD) in pan-cancer with the same protein expression trend as the gene expression (Figures 1F–J). The above results suggest that changes in LAMB3 expression in cancer tissues may be involved in the process of cancer cell development.

### 3.2 Correlation between LAMB3 and molecular and immune subtypes of pan-cancer

**FIGURE 2:**
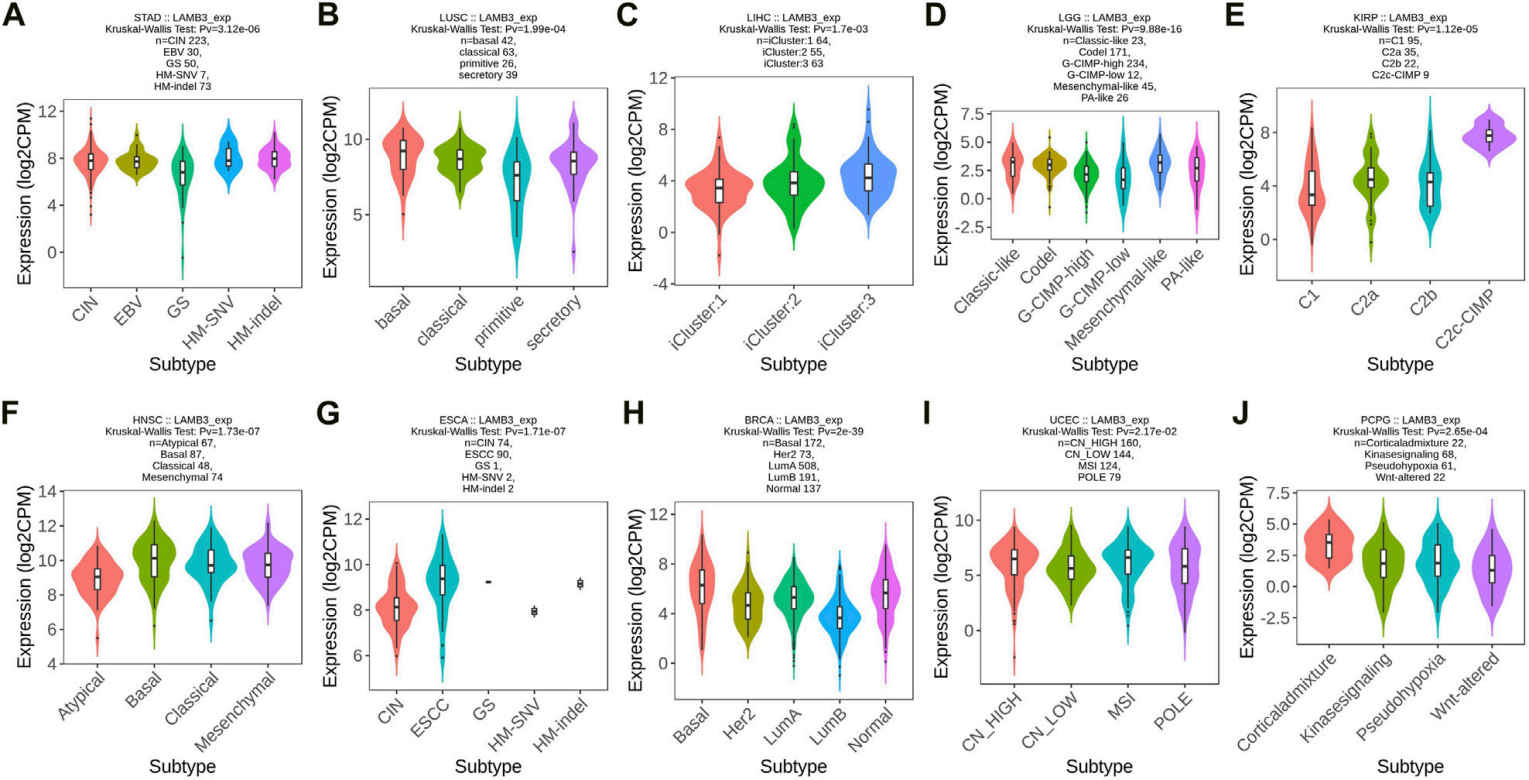
Molecular subtype expression of LAMB3 in pan-cancer. **(A)**. STAD; **(B)**. LUSC; **(C)**. LIHC; **(D)**. LGG; **(E)**. KIRP; **(F)**. HNSC; **(G)**. ESCA; **(H)**. BRCA; **(I)**. UEEC; **(J)**. PCPG.

The correlation between LAMB3 differential expression and pan-cancer molecular subtype is explored from the TISIDB database. LAMB3 was found to be expressed differently in 10 different molecular subtypes, including STAD, LUSC, LIHC, LGG, KIRP, HNSC, ESCA, BRCA, UEEC, and PCPG (Figures 2A–J). Molecular subtypes vary from cancer to cancer. In the case of STAD, LAMB3 has the highest expression in the CIN subtype (Figure 2A). For LUSC, LAMB 3 is most expressed in the basal subtype (Figure 2B). But LAMB3 is most abundant in the Corticaladmixture molecular subtype of PCPG (Figure 2J).

**FIGURE 3:**
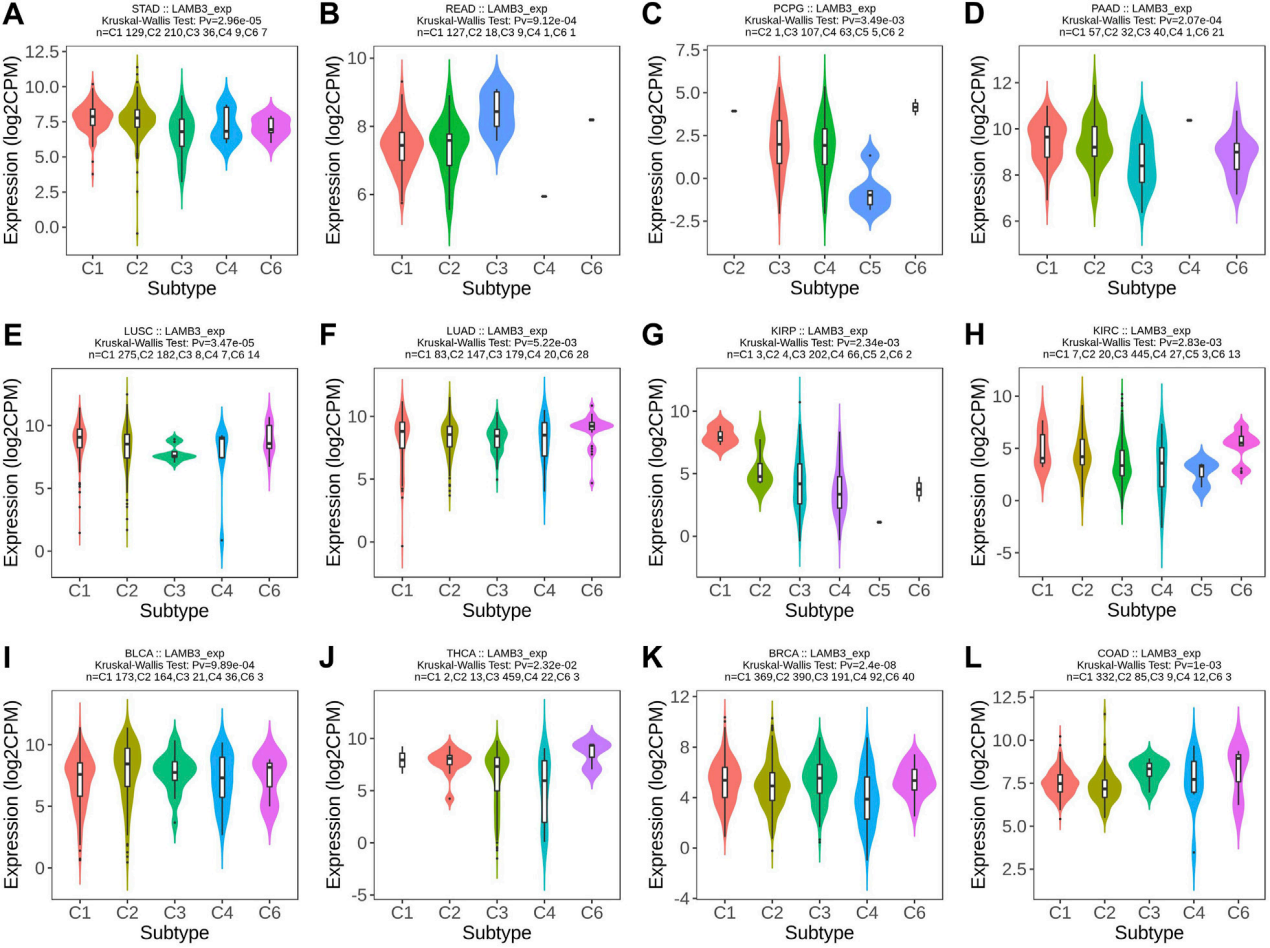
Immunosubtype expression of LAMB3 in pan-cancer. **(A)** STAD; **(B)** READ; **(C)** PCPG; **(D)** PAAD; **(E)** LUSC; **(F)** LUAD; **(G)** KIRP; **(H)** KIRC; **(I)** BLCA; **(J)** THCA; **(K)** BRCA; **(L)**. COAD.

At the same time, we observed that the expression of LAMB3 was significantly correlated with the different immune subtypes of 20 cancer types (C1: wound healing, C2: IFN-gamma dominance, C3: inflammatory, C4: lymphocyte depletion, C5: immune quiet, C6: TGF-b dominance). Cancers containing five or more immune subtypes were STAD, READ, PCPG, PAAD, LUSC, LUAD, KIRP, KIRC, BLCA, THCA, BRCA, and COAD (Figure 3). In addition to this, KICH, PRAD, TGCT, and UVM also have several different immune subtypes (Supplementary Figure S1).

### 3.3 Diagnostic value of LAMB3 in pan-cancer

**FIGURE 4:**
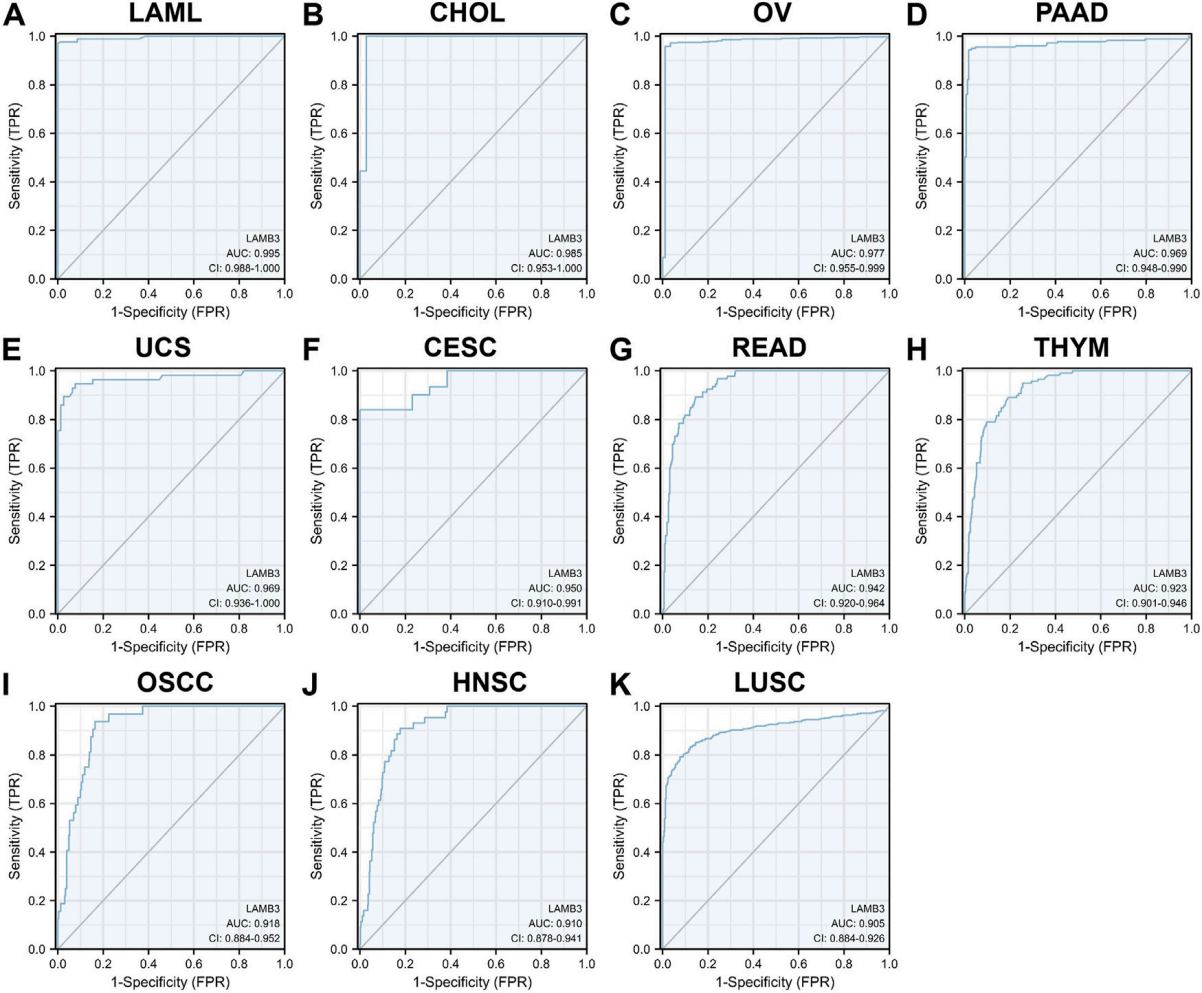
ROC curves of LAMB3 in pan-cancer. **(A)** LAML; **(B)** CHOL; **(C)**OV; **(D)** PAAD; **(E)** UCS; **(F)** CESC; **(G)** READ; **(H)** THYM; **(I)**OSCC; **(J)** HNSC; **(K)** LUAD.

The ROC curve was used to evaluate the diagnostic value of LAMB3 in pan-cancer. The results showed that LAMB3 was extremely accurate (AUC>0.9) in predicting 11 cancer types, including LAML (AUC = 0.995), CHOL (AUC = 0.985), OV (AUC = 0.977), PAAD (AUC = 0.969), UCS (AUC = 0.969), CESC (AUC = 0.950), READ (AUC = 0.942), THYM (AUC = 0.923), OSCC (AUC = 0.918), HNSC (AUC = 0.910), and LUAD (AUC = 0.905) (Figure 4). Meanwhile, LAMB3 had some accuracy (AUC>0.7) in predicting 11 other cancers, including COADREAD, DLBC, ESAD, ESCA, GBM, GBMLGG, KICH, LGG, LUADLUSC, PRAD, SKCM, THCA, and UCEC (Supplementary Figure S2). Indicates that this gene is a potential predictive marker in pan-cancer.

### 3.4 LAMB3 has prognostic relevance in cancer

**FIGURE 5:**
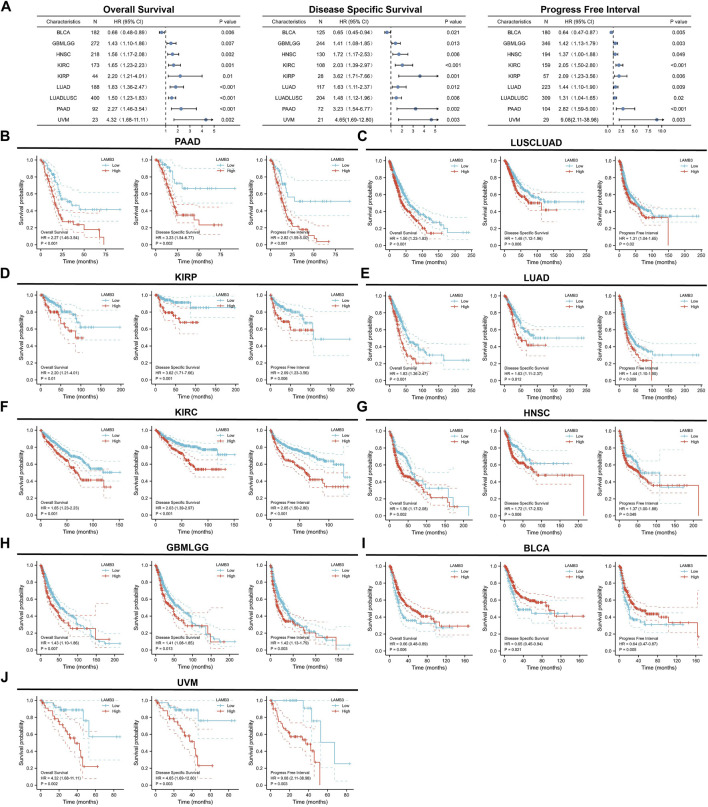
Prognostic KM curves and forest plots of LAMB3 in pan-cancer. **(A)**. Forest plots of LAMB3 in pan-cancer prognosis (OS, DSS and PFI). **(B)**. Prognostic KM curves of LAMB3 gene expression in PAAD; **(C)**. Prognostic KM curves of LAMB3 gene expression in LUSCLUAD; **(D)**. Prognostic KM curves of LAMB3 gene expression in KIRP. **(E)**. Prognostic KM curves of LAMB3 gene expression in LUAD; **(F)**. Prognostic KM curves of LAMB3 gene expression in KIRC; **(G)**. Prognostic KM curves of LAMB3 gene expression in HNSC; **(H)**. Prognostic KM curves of LAMB3 gene expression in GBMLGG. **(I)**. Prognostic KM curves of LAMB3 gene expression in BLCA. **(J)**. Prognostic KM curves of LAMB3 gene expression in UVM.

It is critical to investigate the relationship between LAMB3 expression levels and prognosis, and we performed a survival correlation analysis for each malignancy, including OS, DSS, and PFI. Through forest diagrams, we showed tumors whose LAMB3 expression levels were significantly correlated with OS, DSS, and PFI, including PAAD, LUSCLUAD, KIRP, LUAD, KIRC, HNSC, GBMLGG, BLCA, and UVM (Figure 5A). For PAAD, Cox regression results show that the higher the LAMB3 expression, the worse the prognosis (Figure 5B). This included OS (HR = 2.27, 95%CI: 1.46–3.54, *p* < 0.001), PFI (HR = 2.82, 95% CI: 1.59–5.00, *p* < 0.001), and DSS (HR = 3.23, 95% CI: 1.54–6.77, *p* = 0.002). Higher LAMB3 expression was linked to a worse prognosis for LUSCLUAD, according to Cox regression results (Figure 5C). This included OS (HR = 1.50, 95% CI: 1.23–1.83, *p* < 0.001), PFI (HR = 1.31, 95% CI: 1.04–1.65, *p* = 0.02), and DSS (HR = 1.48, 95% CI: 1.12–1.96, *p* = 0.006). For KIRC, Cox regression results show that the higher the LAMB3 expression, the worse the prognosis (Figure 5D). This included OS (HR = 2.20, 95% CI: 1.21–4.01, *p* = 0.01), PFI (HR = 2.09, 95% CI: 1.23–3.56,*p* = 0.006), and DSS (HR = 3.62, 95% CI: 1.71–7.66, *p* = 0.001). Higher LAMB3 expression was linked to a worse prognosis for LUAD, according to Cox regression results (Figure 5E). This included OS (HR = 1.83, 95% CI: 1.36–2.47, *p* < 0.001), PFI (HR = 1.44, 95% CI: 1.10–1.90, *p* = 0.009), and DSS (HR = 1.63, 95% CI: 1.11–2.37, *p* = 0.012). For KIRC, Cox regression results show that the higher the LAMB3 expression, the worse the prognosis (Figure 5F). This included OS (HR = 1.65, 95% CI: 1.23–2.23, *p* = 0.001), PFI (HR = 2.05, 95% CI: 1.50–2.80, *p* < 0.001), and DSS (HR = 2.03, 95% CI: 1.39–2.97, *p* < 0.001). Higher LAMB3 expression was linked to a worse prognosis for HNSC, according to Cox regression results (Figure 5G). This included OS (HR = 1.56, 95% CI: 1.17–2.08, *p* = 0.002), PFI (HR = 1.37, 95% CI: 1.00–1.88, *p* = 0.049), and DSS (HR = 1.72, 95% CI: 1.17–2.53, *p* = 0.006). For GBMLGG, Cox regression results show that the higher the LAMB3 expression, the worse the prognosis (Figure 5H). This included OS (HR = 1.43, 95% CI: 1.10–1.86, *p* = 0.007), PFI (HR = 1.42, 95% CI: 1.13–1.79,*p* = 0.003), and DSS (HR = 1.41, 95% CI: 1.08–1.85, *p* = 0.013). Higher LAMB3 expression was linked to a better prognosis for BLCA, according to Cox regression results (Figure 5I). This included OS (HR = 0.66, 95% CI: 0.48–0.89, *p* = 0.006), PFI (HR = 0.64, 95% CI: 0.47–0.87, *p* = 0.005), and DSS (HR = 0.65, 95% CI: 0.45–0.94, *p* = 0.021). For UVM, Cox regression results show that the higher the LAMB3 expression, the worse the prognosis (Figure 5J). This included OS (HR = 4.32, 95% CI: 1.68–11.11, *p* = 0.002), PFI (HR = 9.08, 95% CI: 2.11–38.98, *p* = 0.003), and DSS (HR = 4.65, 95% CI: 1.69–12.80, *p* = 0.003). Additionally, Cox regression results in COAD, KICH, LIHC, OS, OV, PRAD, READ, SKCM, STAD, TGCT, THYM, CESC, GBM, SARC, BRCA, UCEC, and UCS reveal that some aspects of the prognosis for cancer are correlated with the level of LAMB3 expression.

### 3.5 Correlation of LAMB3 expression levels with immune cell infiltration, ICP gene, TMB, and MSI in pan-cancer

**FIGURE 6:**
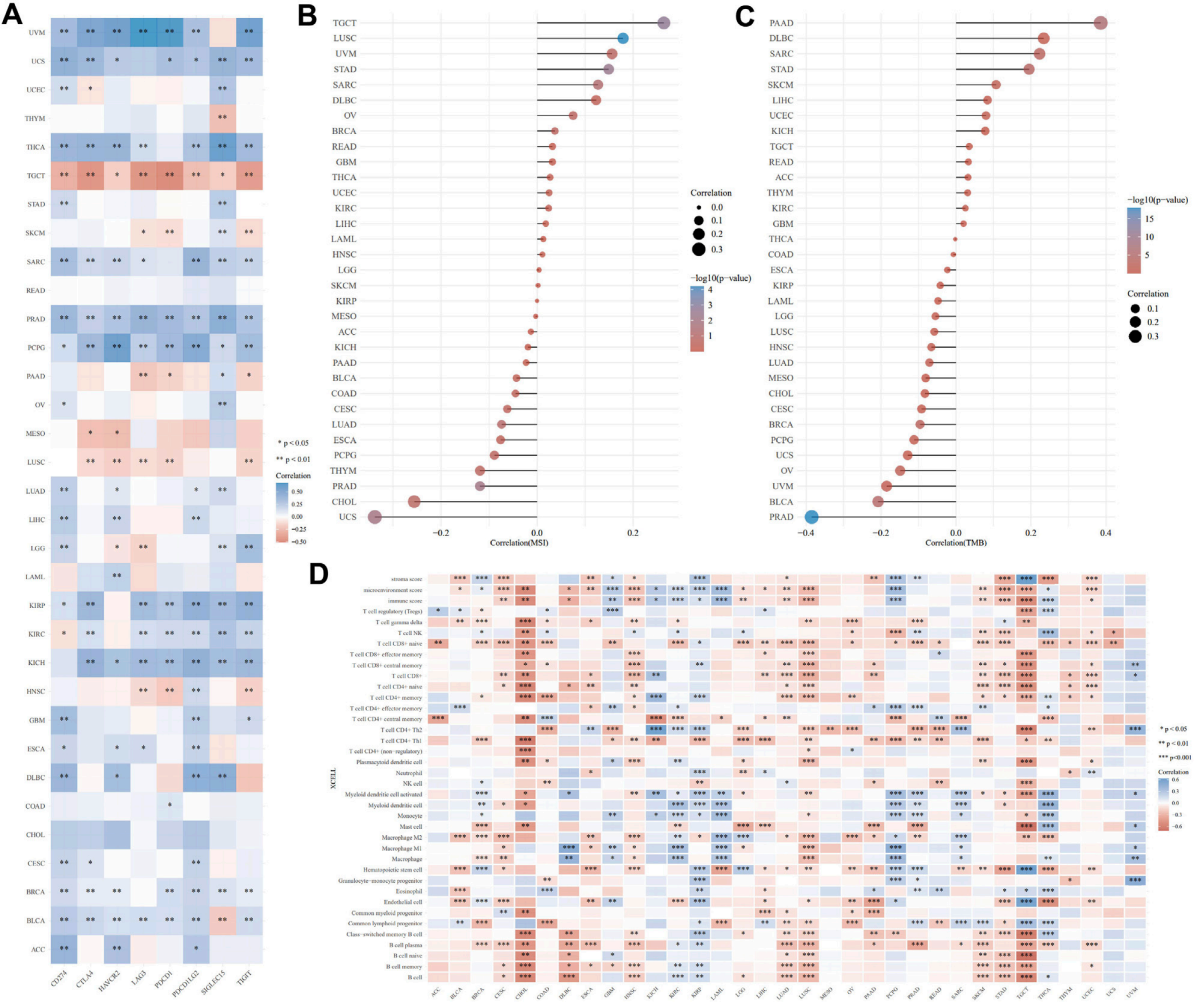
Immune correlation analysis of LAMB3 expression levels with pan-carcinoma. **(A)**. Correlation of LAMB3 expression with ICP genes; **(B)**. Correlation of LAMB3 expression levels with MSI; **(C)**. Correlation of LAMB3 expression levels with TMB; **(D)**. Correlation of LAMB3 expression levels with immune-related cellular infiltration levels.

An emerging method of treating tumors is immunotherapy. ICP genes have been shown to have a significant impact on immunotherapy and the infiltration of immune cells. We originally investigated the relationship of LAMB3 expression with ICP qualities in human malignant growths and the capability of LAMB3 in immunotherapy (Figure 6A). The LAMB3 expression of TGCT, SKCM, PAAD, MESO, LUSC, and HNSC in 8 ICP gene transcripts was highly positively correlated with the immune checkpoint gene. Immunotherapy that targets ICP genes may have a favorable therapeutic effect if LAMB3 expression is high. LAMB3 expression was negatively correlated with ICP genes in UVM, USC, THCA, STAD, SARC, PRAD, PCPG, OV, LUAD, KIRP, KICH, GBM, ESCA, DLBC, CESE, BRCA, BLCA, and ACC, suggesting that high LAMB3 expression may indicate poor outcomes from immunotherapeutics targeting ICP genes.

The relationship between TMB and MSI, which are both intrinsically linked to the sensitivity of immune checkpoint inhibitors, and LAMB3 expression levels was the next topic of our investigation. MSI has a significant positive correlation with TGCT, LUSC, UVM, STAD, SARC, DLBC, OV, BRCA, READ, GBM, THCA, UCEC, KIRC, LIHC, LAML, HNSC, LGG, and SKCM, according to the MSI correlation results. MSI has a significant negative correlation with KIRP, MESO, ACC, KICH, PAAD, BLCA, COAD, CESC, LUAD, ESCA, PCPG, THYM, PRAD, CHOL, and UCS (Figure 6B). TMB has a significant positive correlation with PAAD, DLBC, SARC, STAD, SKCM, LIHC, UCEC, KICH, TGCT, READ, ACC, THYM, KIRC, and GBM, according to the TMB correlation results. MSI has a significant negative correlation with THCA, COAD, ESCA, KIRP, LAML, LGG, LUSC, HNSC, LUAD, MESO, CHOL, CESC, BRCA, PCPG, UCS, OV, UVM, BLCA, and PRAD (Figure 6C).

Following that, we investigated the connection between the levels of infiltration of 26 immune-related cells and the expression levels of LAMB3. Immune cell infiltration levels were significantly correlated with LAMB3 expression in most types of cancer (Figure 6D). LAMB3 expression was strongly correlated with the degree of immune cell infiltration in CESE, CHOL, COAD, DLBC, ESCA, HNSC, LGG, LIHC, LUAD, LUSC, PAAD, SKCM, STAD, TGCT, and UCEC. In GBM, KIRP, KIRC, THCA, UVM, and PCPG, LAMB3 expression was highly negatively correlated with the degree of immune cell infiltration. In other tumors, there was a positive and a negative correlation with the level of immune cell infiltration. As a result, we hypothesize that LAMB3 could be a novel immunotherapy target or a pan-cancer biomarker that could predict the response to immunotherapy or produce promising therapeutic outcomes.

### 3.6 PPI network, GO and KEGG enrichment analysis of LAMB3 interaction protein

**FIGURE 7:**
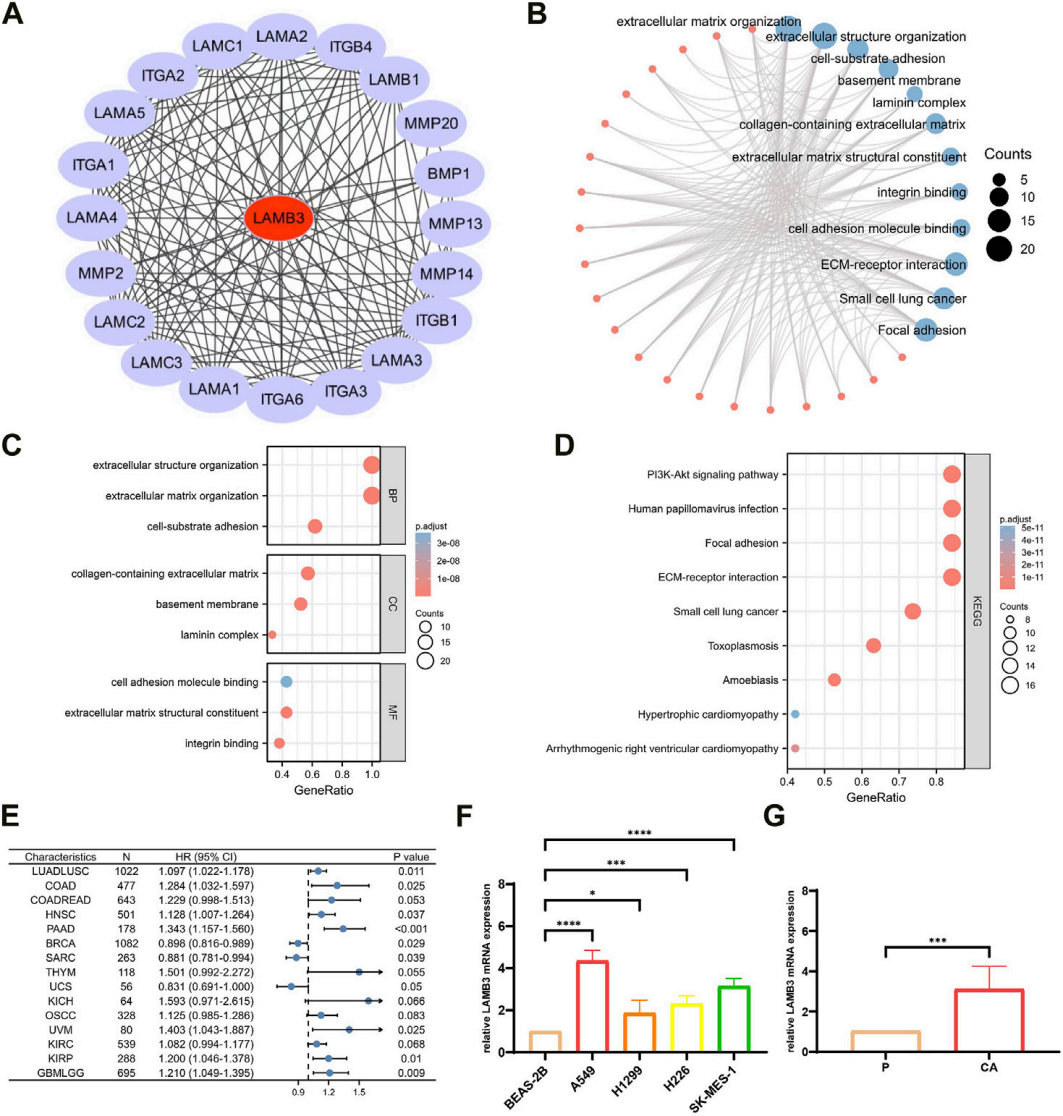
Enrichment analysis of LAMB3 gene expression and reciprocal proteins. **(A)**. PPI network of 20 target binding proteins of LAMB3; **(B)**. visual network of GO and KEGG analysis; **(C)**. GO analysis; **(D)**. KEGG analysis; **(E)**. forest plot of multifactorial COX regression analysis of LAMB3 in pan-cancer; **(F)**. four lung cancer (squamous lung cancer/lung adenocarcinoma) cell lines *versus* normal lung epithelial cells for LAMB3 mRNA expression; **(G)**. LAMB3 mRNA expression in cancerous *versus* paraneoplastic tissues of lung cancer patients.

20 proteins that interact with LAMB3 proteins were found using the STRING database (Figure 7A). The 20 target-binding proteins were then subjected to GO enrichment analysis (Figure 7B). In this case, the primary biological process (BP) contains extracellular matrix organization, extracellular structure organization, and cell-substrate adhesion. The cellular component (CC) was mainly enriched in the basement membrane, laminin complex, and collagen-containing extracellular matrix. The molecular function (MF) has mainly participated in extracellular matrix structural constituent, integrin binding and cell adhesion molecule binding (Figure 7C). According to the results of the KEGG pathway enrichment, it is mostly linked to ECM-receptor interaction, Small cell lung cancer, Focal adhesion, Human papillomavirus infection, PI3K-Akt signaling pathway, Toxoplasmosis, Amoebiasis, Arrhythmogenic right ventricular cardiomyopathy and Hypertrophic cardiomyopathy (Figure 7D) (Supplementary Table S2).

In conclusion, our pan-cancer analysis revealed that LAMB3 is an independent risk factor for multiple cancers and is closely linked to their development and prognosis (Figure 7E) (Supplementary Table S3). In particular, LAMB3 expression levels were found to have a strong correlation with all aspects of lung cancer. Using lung cancer as an example, we investigated this further in this section. LAMB3 gene expression levels were higher in 8 cases of lung cancer (Figure 7F) and 4 lung cancer cell lines (lung scale/lung adenocarcinoma) (Figure 7G) than in paracarcinous tissue or normal lung epithelial cells. Trends in gene expression match predictions made for pan-cancer.

### 3.7 Correlation between expression of LAMB3 gene and lung cancer

**FIGURE 8:**
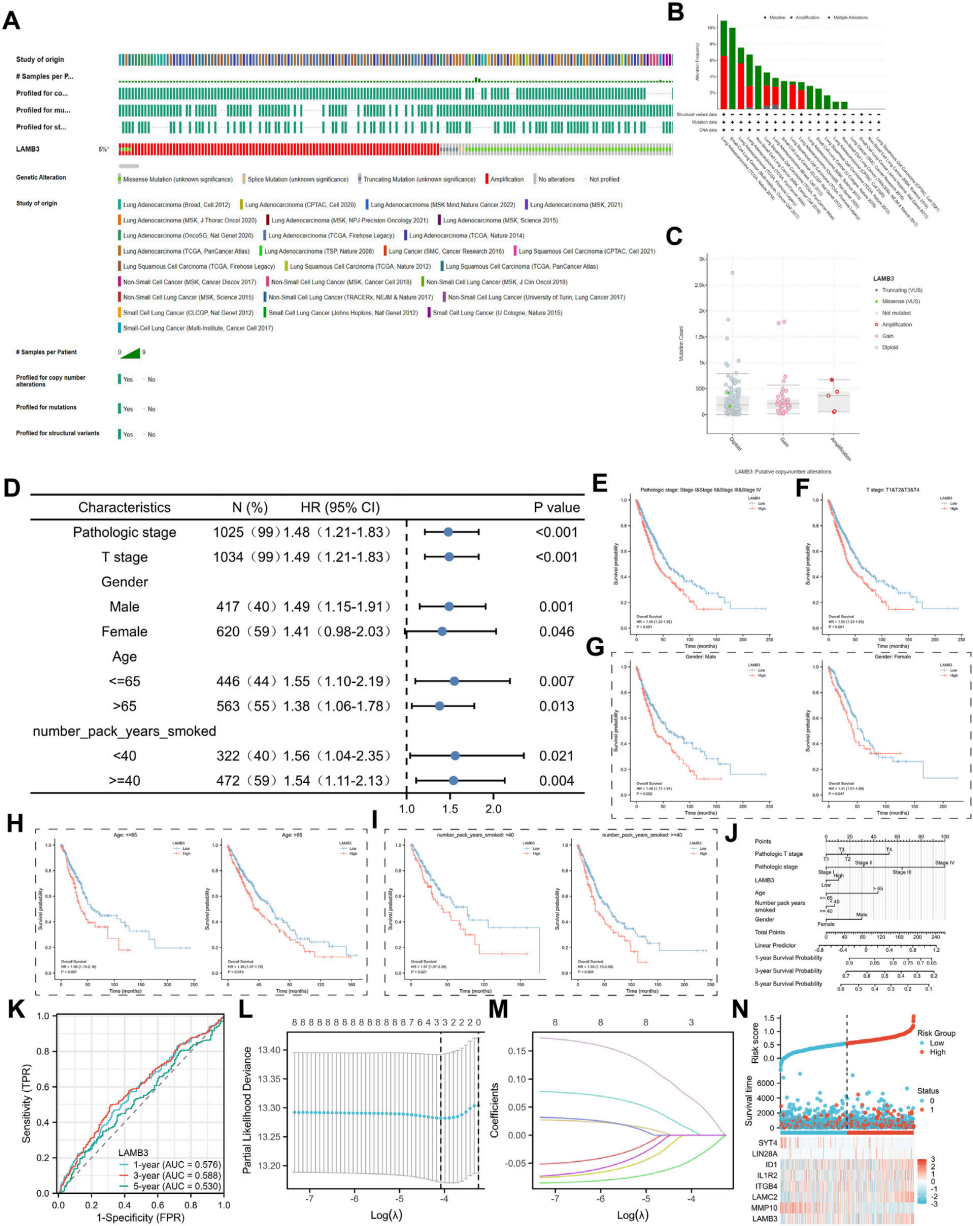
LAMB3 genomic alterations in lung cancer and correlation between LAMB3 and prognosis in different clinical subgroups of lung cancer. **(A)**. OncoPrint of LAMB3 gene alterations in cancer cohort; **(B)**. Detailed information on the types of LAMB3 gene alterations in lung cancer cohort; **(C)** *. Major* types of LAMB3 gene alterations in lung cancer; **(D)**. Forest plot of LAMB3 in clinical subgroups with lung cancer; **(E)**. Prognostic correlation between LAMB3 expression in pathologic stage of lung cancer; **(F)**. Prognostic correlation between LAMB3 expression in T stage of lung cancer patients; **(G)**. Prognostic correlation between LAMB3 expression in gender of lung cancer patients; **(H)**. Prognostic correlation between LAMB3 expression in age of lung cancer patients; **(I)**. Prognostic correlation between LAMB3 expression in number_pack_years_smoked of lung cancer patients; II. pack_years_smoked in lung cancer patients with prognostic correlation; **(J)**. 1, 3, and 5-year nomogram for predicting OS of lung cancer; **(K)**. The signature is shown by the time-dependent ROC curve for predicting 1, 3, and 5-year survival; **(L)**. Ten-time cross-validation for tuning parameter selection in the LASSO model; **(M)**. LASSO coefficient profiles; **(N)**. The risk score, survival status, and heat map of eight genes in patients with lung cancer.

LAMB3 genomic alterations in lung cancer were explored via the cBioPortal website. The findings demonstrated that 5% of LAMB3 genomic changes occurred (Figure 8A). Changes in gene expression are brought about by a variety of LAMB3 gene alterations, including diploid, gain, and amplification (Figure 8B). Copy number variants (CNV) were positive in half of the sequencing results (Figure 8C). The expression of LAMB3 in lung cancers with various clinical features was then further investigated (Supplementary Table S4). We have summarised the above table in the form of a forest diagram (Figure 8D). Pathologic stage (Figure 8E), T stage (Figure 8F), Gender (Figure 8G), Age (Figure 8H) and number_pack_years_smoked (Figure 8I) have worse prognosis when LAMB3 high expression. In addition, we developed a nomogram model with the parameters of LAMB3 expression levels, age, gender, T stage, and pathologic stage (Figure 8J).

These factors were identified as highly significant prognostic predictors based on multivariate Cox regression analysis. Columnar plots showed significantly higher clinical value in predicting the probability of survival at 1, 3, and 5 years in patients with lung cancer carcinoma. We further used ROC curves to assess the accuracy of LAMB3 in predicting OS in lung cancer patients. As shown in Figure 8K, the AUC values of 0.576, 0.588, and 0.530 at 1, 3, and 5 years, respectively, demonstrated the accuracy of LAMB3 in predicting patient prognosis. We performed further analysis of the co-expressed genes of LAMB3 in lung cancer. Subsequent univariate Cox regression analysis yielded eight genes significantly associated with prognosis (Supplementary Table S6). Further, LASSO regression analysis revealed that the above genes were considered risk factors affecting the prognosis of lung cancer patients (Figures 8L–N).

### 3.8 Correlation of LAMB3 expression levels with immune cell infiltration in lung cancer

**FIGURE 9:**
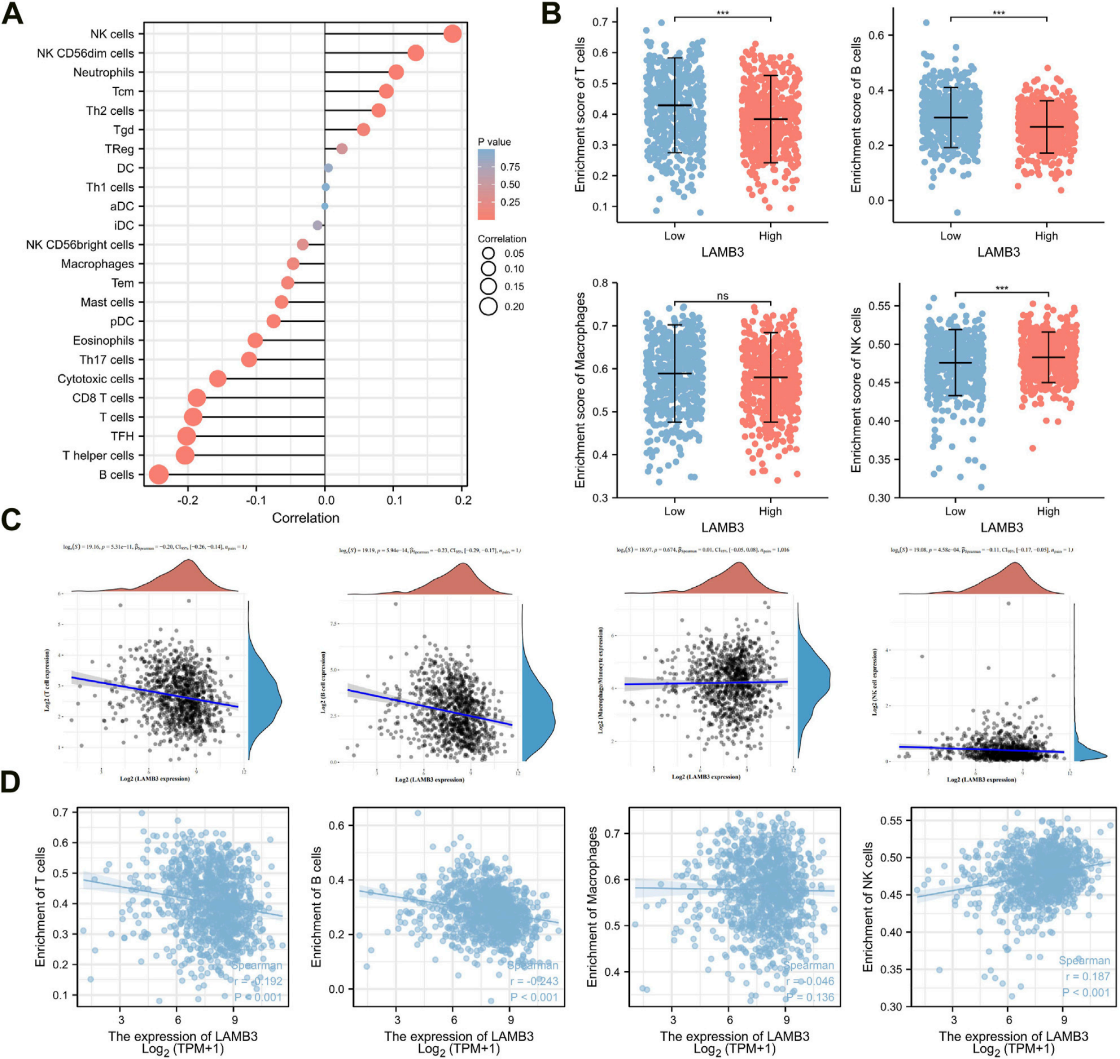
LAMB3 Expression levels are associated with immune cell infiltration in lung cancer. **(A)**. Lollipop diagram of LAMB3 expression infiltrated with immune cells in lung cancer; A grouping map of **(B)**. T cells, B cells, Macrophages and NK cells with LAMB3 gene expression; Correlation analysis of **(C)**. T cells, B cells, Macrophages and NK cells with LAMB3 gene expression (EPIC analysis); Correlation analysis of **(D)**. T cells, B cells, Macrophages and NK cells with LAMB3 gene expression (ssGSEA).

We investigated the connection between the amount of infiltration of 26 immune-related cells and the level of LAMB3 expression in lung cancer. LAMB3 gene expression was positively correlated with Natural killing (NK) cells, NK CD56dim cells, Neutrophils, Tcm, Th2 cells, Tgd, Treg, DC, and Th1 cells. LAMB3 gene expression was negatively correlated with aDC, B cells, CD8 T cells, Cytotoxic cells, Eosinophils, iDC, Macrophages, Mast cells, NK CD56bright cells, pDC, T cells, T helper cells, Tem, TFH and Th17 cells (Figure 9A). After that, we carried out the connection between the expression of LAMB3 genes on T cells, B cells, macrophages, NK cells, and B cells in the immune system. In group comparisons, we found that the high-expression LAMB3 group had a lower number of infiltrating T cells, B cells, and macrophages and a higher number of infiltrating NK cells (Figure 9B). The Spearman method was used to investigate the relationship between immune cell infiltration and LAMB3 expression. T cells, B cells, macrophages, and NK cells all have a negative correlation with LAMB3, according to the EPIC method analysis (Figure 9C). Using the immune infiltration algorithm (ssGSEA), we also discovered that LAMB3 was negatively correlated with T cells, B cells, and macrophages. In contrast, NK cells demonstrated a positive correlation with LAMB3, which is in line with previous findings regarding the infiltration of immune cells (Figure 9D).

### 3.9 The methylation status of the LAMB3 gene is closely associated with lung cancer patients

**FIGURE 10:**
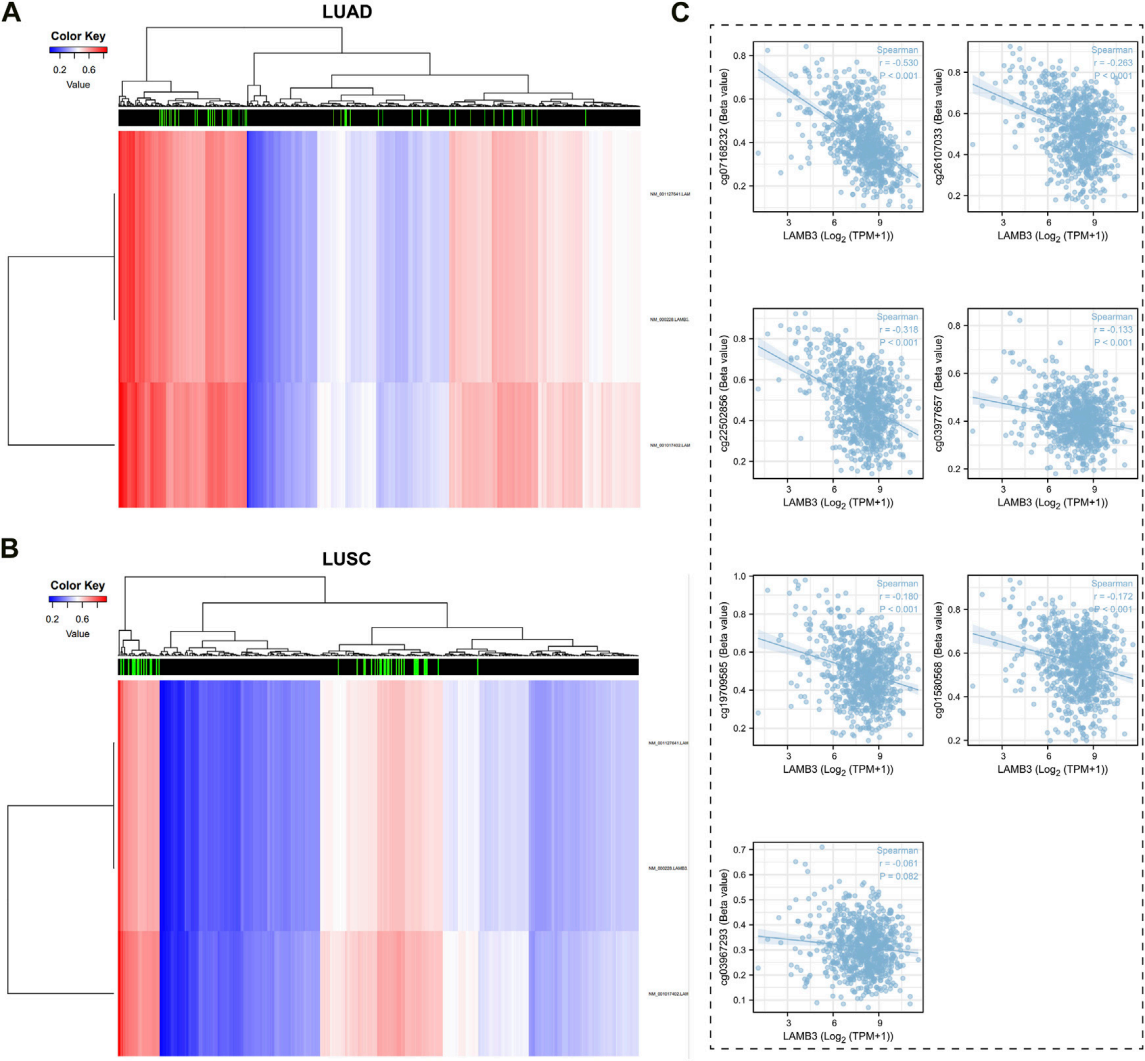
Methylation status of LAMB3 genes in patients with lung cancer. **(A,B)**. DNA methylation levels in patients with lung cancer and heat map results of CpG islands in LAMB3 genes; **(C)**. Analysis of CpG Island- LAMB3 Expression Correlation in Methylation.

The DiseaseMeth tool was used to look at the relationship between the levels of DNA methylation and CpG islands in the LAMB3 gene (Figures 10A, B). All seven methylated CpG islands had a negative correlation with LAMB3 expression, as demonstrated by the correlation results, including cg07168232, cg26107033, cg22502856, cg03977657, cg19709585, cg01580568, and cg03967293 (Figure 10C).

### 3.10 Analysis of LAMB3 co-expression gene and functional enrichment in lung cancer

**FIGURE 11:**
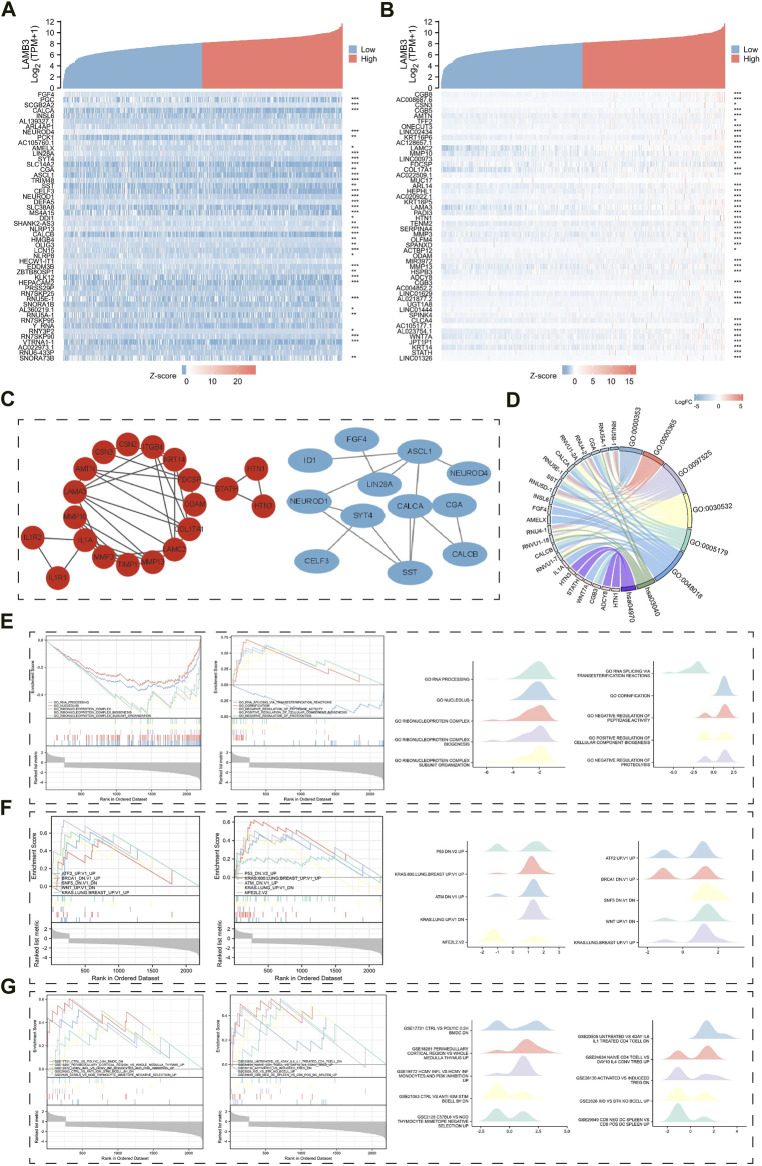
Results of LAMB3 co-expression genes in lung cancer and their functional enrichment analysis. **(A)**. Gene co-expression heat map of the top 50 genes negatively associated with LAMB3 in lung cancer; **(B)**. Gene co-expression heat map of the top 50 genes positively associated with LAMB3 in lung cancer; **(C)**. PPI network map of LAMB3 co-expressed genes (red: positively associated genes, blue: negatively associated genes); **(D)**. Chord plot of GO/KEGG analysis of LAMB3 co-expressed genes; **(E)**. Visualization of GSEA analysis of LAMB3 co-expressed genes and gene ontology; **(F)**. Visualization of GSEA analysis of LAMB3 co-expressed genes and oncogenic signatures; **(G)**. GSEA analysis of LAMB3 co-expressed genes and gene ontology. The results of GSEA analysis and Immunologic signatures of LAMB3 co-expressed genes.

In a heat map, we explored the top 50 co-expressed genes that were either positively or negatively associated with LAMB3 expression in lung cancer (Figures 11A, B). Following the established thresholds, 955 DEGs, including 873 downregulated genes and 82 upregulated genes, were gathered (Supplementary Table S5). We searched for the hub gene by performing a PPI network map of the respective genes that were upregulated and downregulated (Figure 11C). The analysis of the GO and KEGG enrichment of DEGs indicates that the BP of the LAMB3 co-expressed differential gene primarily includes the formation of quadruple SL/U4/U5/U6 snRNP and mRNA trans-splicing, via spliceosome. CC is mainly involved in spliceosomal snRNP complex and small nuclear ribonucleoprotein complex, *etc.* MF primarily participates in hormone activity and receptor-ligand activity, *etc.* KEGG pathway enrichment was mainly associated with Spliceosome, Salivary secretion, and RNA transport (Figure 11D).

Finally, we used GSEA enrichment analysis to investigate the significance of LAMB3-related DEGs in terms of gene ontology, oncogenic signatures, and immunological signatures. The top ten data are mainly shown in the images (Supplementary Tables S6–S8). Gene ontology is mainly related to GO_RNA_PROCESSING, GO_NUCLEOLUS, GO_RIBONUCLEOPROTEIN_COMPLEX, GO_RIBONUCLEOPROTEIN_COMPLEX_BIOGENESIS, and GO_RIBONUCLEOPROTEIN_COMPLEX_SUBUNIT_ORGANIZATION (Figure 11E). Oncogenic signatures are mainly related to ATF2_UP.V1_UP, BRCA1_DN.V1_UP, SNF5_DN.V1_DN, WNT_UP.V1_DN” and KRAS. LUNG.BREAST_UP.V1_UP (Figure 11F). Immunologic signatures is mainly related to CTRL_VS_POLYIC_0.5H_BMDC_DN, CTRL_VS_ANTI_IGM_STIM_BCELL_8H_DN, PERIMEDULLARY_CORTICAL_REGION_VS_WHOLE_MEDULLA_THYMUS_UP, HCMV_INFL_VS_HCMV_INF_MONOCYTES_AND_PI3K_INHIBITION_UP and C57BL6_VS_NOD_THYMOCYTE_MIMETOPE_NEGATIVE_SELECTION_UP (Figure 11G). The results show that the gene is closely related to gene ontology, oncogenic signatures, and immunological signatures. This also indicates that LAMB3 can serve as potential tumor biomarkers and therapeutic targets.

### 3.11 LAMB3 affects the biological function of lung cancer cells

**FIGURE 12:**
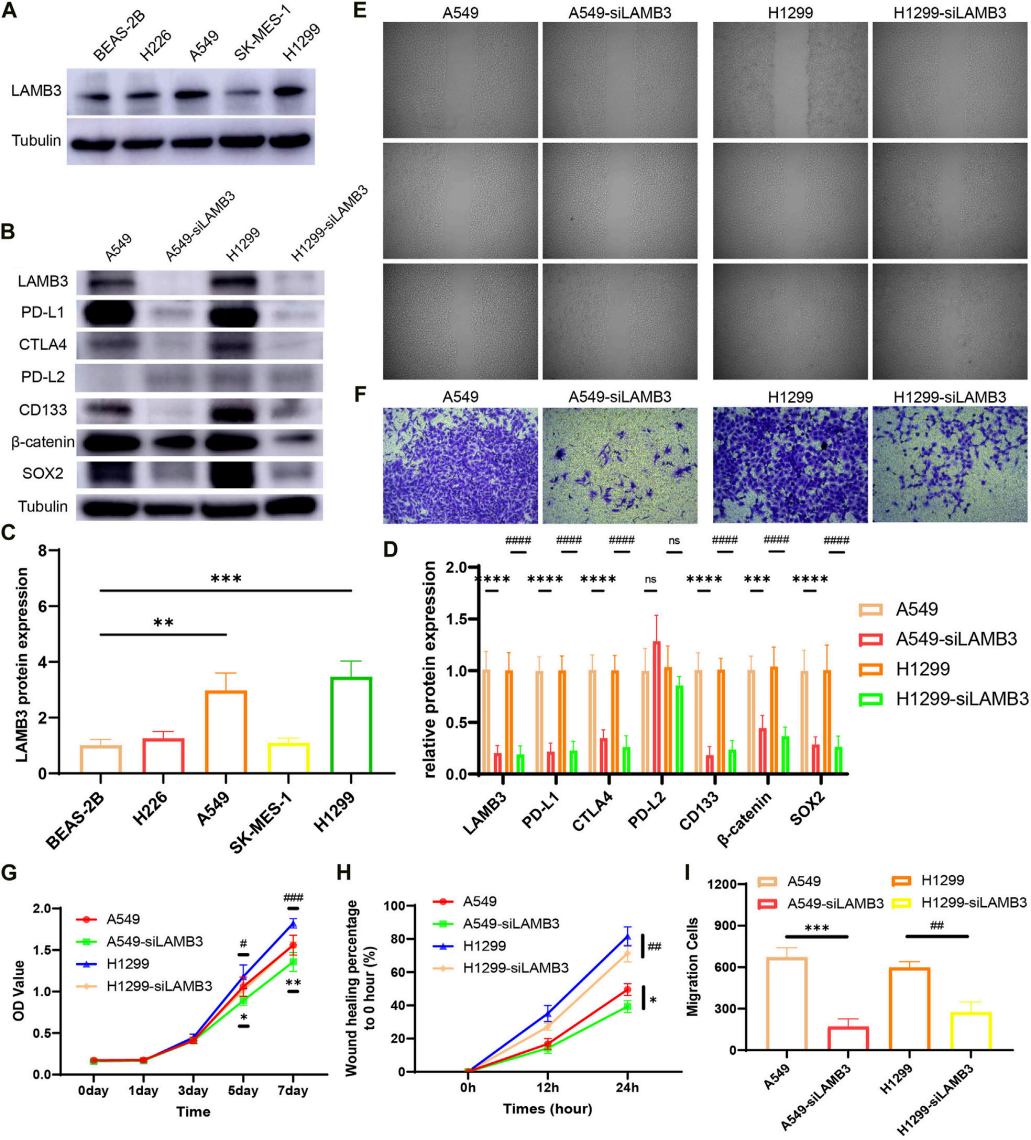
LAMB3 promotes the malignant behavior of lung cancer cells. **(A,C)**. Protein expression of LAMB3 in normal lung epithelial cells and lung cancer cell lines and their statistical results; **(B,D)**. Knockdown of LAMB3 on related protein expression in A549 and H1299 cells and their statistical results; **(E,H)**. Cell scratching results of the 4 cell lines and their statistical analysis; **(F,I)**. Invasion assay results of the 4 cell lines and their statistical analysis; **(G)**. Results of CCK8 cell proliferation assay for the 4 cell strains. (*: A549 vs*.* A549-siLAMB3, #: H1299 vs*.* H1299-siLAMB3).

Experiments showed that the protein expression level of LAMB3 in lung cancer cell lines A549 and H1299 was significantly higher than that in normal lung epithelial cells BEAS-2B (Figures 12A, C). In a follow-up experiment, WB demonstrated that we successfully knocked down LAMB3 expression in the above two cell lines using siRNA (Figures 12B, D).

The effect of LAMB3 on the migration ability of lung cancer cell lines A549, A549-siLAMB3, H1299, and H1299-siLAMB3 was analyzed in wound healing assays. The results showed that inhibition of LAMB3 expression significantly inhibited the migratory ability of lung cancer cells, which was statistically significant (Figures 12E, H). We also examined the effect of knocking down LAMB3 on the invasive ability of cells. The results showed that the invasive ability of both cell lines was equally inhibited after the knockdown of LAMB3 (Figures 12F, I). Finally, we found that the proliferation ability of each cell line was significantly diminished after the knockdown of LAMB3 (Figure 12G).

Finally, we performed a preliminary exploration of the mechanism based on the previous bioinformatics-related results. We found that inhibition of LAMB3 expression significantly suppressed the expression of PD-L1 and CTLA4 in cells, but there was no statistically significant difference in PD-L2 expression. Notably, both PD-L1 and CTLA4 are closely associated with T cell and tumor immunity. Meanwhile, inhibition of LAMB3 expression decreased the expression of cell stemness-related gene CD133 and β-catenin-related signaling pathway (Figures 12B, D).

## 4 Discussion

Cancer is a significant risk factor for human health worldwide. Early detection and destruction of tumor cells are important conditions for improving the prognosis of cancer patients (Li et al., 2022). However, the most common cancer treatments, such as radiotherapy, chemotherapy, and surgical resection, have limited therapeutic effects. At the same time, the aggressiveness, metastasis, and drug resistance of tumors pose a similar challenge to cancer treatment (Li et al., 2017; Bahar et al., 2020). It has been demonstrated that LAMB3 plays a significant role in the progression of some types of cancer, and its function is closely associated with attachment, migration, and interaction with other components of the extracellular matrix (Huang et al., 2019). Abnormal promoter methylation and the silencing of LAMB3 are strongly correlated with the stage, size, and aggressiveness of breast cancer tumors (Sathyanarayana et al., 2003; Sundqvist et al., 2020). It has also been shown that LAMB3 is associated with diagnosis, prognosis, and the immune microenvironment in pancreatic cancer (Yang et al., 2020; Chen et al., 2022). Additionally, in prostate, thyroid, colorectal, and other cancers, LAMB3 may aid in the growth of tumors (Reis et al., 2013; Jung et al., 2018; Zhu et al., 2020). As a result, LAMB3 has the potential to be a novel pan-cancer biomarker, a potential therapeutic target, and a player in tumor formation and progression. By examining the relationship between LAMB3 expression and prognosis, immune microenvironment, and DNA methylation in cancer patients, we used a variety of bioinformatics to investigate the potential role of LAMB3 in pan-cancer.

To begin, we examined the levels of LAMB3 gene and protein expression in normal and cancerous tissues using the GTEx, TCGA, BioGPS, and HPA databases. The findings demonstrated that the majority of cancer types exhibited a significant upward trend in the expression of LAMB3. This is in line with the trend that other researchers have observed in cancers like colorectal, thyroid, pancreatic, and head and neck squamous cell carcinomas (Wang et al., 2018; Liu et al., 2019; Zhu et al., 2020; Islam et al., 2021). LAMB3’s high expression is consistent with the expression trends we find in lung cancer cell lines and the tissues of lung cancer patients.

Secondly, we investigated the possibility of LAMB3 functioning as a biomarker for the diagnosis of pan-cancer. The results indicate that LAMB3 has excellent diagnostic value in most cancers. LAMB3 is also an independent risk factor for most cancers. Meanwhile, in the constructed forest plot of LAMB3 and clinicopathological features, high LAMB3 expression in most cancers, including LUSCLUAD, KIRP, KIRC, and HNSC, is associated with a poor prognosis. But there are also a few cancers, such as BLCA, where high LAMB3 expression means a better prognosis. The expression of LAMB3 in the molecular and immune subtypes of pan-carcinoma was then investigated to learn more about its potential mode of action. The findings demonstrated that the most of cancer types had significantly distinct expression patterns of LAMB3 in various immune and molecular subtypes. The above results indicate that LAMB3 is a potential pan-cancer prognostic biomarker, even though expression and prognosis differ in a small number of cancers.

Tumor-infiltrating lymphocytes (TIL) and TMB are important factors in good immunotherapy outcomes and pan-cancer prognosis (Azimi et al., 2012; Steuer and Ramalingam, 2018; Fumet et al., 2020). Our study shows a strong correlation between LAMB3 and TILs. In the majority of cancers, including lung cancer, LAMB3 expression was negatively correlated with T cells, B cells, and macrophages. In most cancers, including lung cancer, LAMB3 expression was positively correlated with NK cells. It has been demonstrated that macrophages, as an essential component of the tumor microenvironment (TME), aid in immune evasion and suppression (Gajewski et al., 2013). NK cells with high expression can improve the capacity of DC cells to present tumor cross antigens (Han et al., 2019). Furthermore, LAMB3 expression was correlated with all marker genes for other cytokines that are known to be immunostimulatory and immunosuppressive, indicating that LAMB3 may play an immune function in lung cancer and other cancers. In addition to confirming that LAMB3 expression is closely linked to the biological processes of immune cells and immune-related molecules in the majority of cancers, our research provides additional insight into the broader suitability of LAMB3 for tumors. The close connection between LAMB3 and TME in human cancers is also demonstrated by the correlation between LAMB3 and TMB and MSI. In outline, the prognosis of cancer patients can be influenced by LAMB3 expression, which is closely linked to the immune infiltration of tumor cells. Immunosuppressive agents may now have a new target in mind with this gene.

We examined the LAMB3 co-expression network at the end. In terms of immunity, we discovered that antigen processing and presentation, B/T cell activation, and immune response regulation are all performed by LAMB3 and its co-expressed genes. We have also found LAMB3 is equally closely related in terms of oncogenic signatures and gene ontology. Furthermore, at the cellular level, we found that reducing LAMB3 expression in lung cancer cell lines A549 and H1299 inhibited the proliferation, migration, and invasive ability of tumor cells. Preliminary mechanistic exploration showed that it can regulate PD-L1 and CTLA4 expression, echoing the above findings that LAMB3 regulates T cell and tumor immunity. CTLA-4 inhibits T cell activation by competing with CD28 receptors to bind to B7 ligands on antigen-presenting cells (Stamper et al., 2001; Fife and Bluestone, 2008). PD-L1 is a ligand of PD-1, and their binding can transmit inhibitory signals in T cells, thereby reducing T cell proliferation and tumor-killing activity (Francisco et al., 2010). Both CTLA-4 and PD-1 are common inhibitory checkpoints on activated T cells and have been found to be the most reliable targets for cancer treatment. The combination of CTLA-4 and PD-1 blockers has a synergistic effect on the activation of anti-tumor immune response (Rotte, 2019). LAMB3 is likely to inhibit T cell infiltration through the above-mentioned targets, thus enabling “immune escape.” Meanwhile, LAMB3 may also promote the malignant behavior of tumor cells by regulating tumor cell stemness and β-catenin-related signaling pathways. Taken together, the above results suggest that LAMB3 has the potential to be a target for anti-cancer immunotherapy.

In summary, we have performed the first comprehensive and systematic analysis of LAMB3 and cross-validated it using different databases, patient samples, and tumor cell lines. The factor was expressed differently between most tumors and normal tissues and revealed the correlation between LAMB3 expression and clinical prognosis, immune microenvironment, and DNA methylation. Although these bioinformatics are carried out by collecting information from various databases, clinical samples, and tumor cell lines, there are some limitations to this study. First, distinct databases contain cancers that are at odds with one another. To better understand LAMB3’s expression and function, additional research in this area would require a larger number of clinical and tumor cell lines. Second, despite the investigation of the gene’s potential signaling pathways and its prognostic and diagnostic value in pan-cancer, more in-depth *in vitro* or *in vivo* experiments to confirm these findings are lacking. Third, despite our findings that immune cell infiltration and associated immune targets in human cancers are closely linked to LAMB3 gene expression, we lack direct evidence that LAMB3 influences prognosis by participating in immune infiltration. The mechanism by which LAMB3 is involved in immune regulation is still unknown, and the effect of LAMB3 on tumor immunity also varies depending on the type of tumor. The mechanisms of related immunotherapy require additional cellular and animal research. In conclusion, further investigation of LAMB3 in lung cancer and its significance in pan-carcinoma diagnosis and prognosis can contribute new perspectives to a comprehensive comprehension of its crucial role in tumor progression. Furthermore, this research provides a comprehensive analytical foundation for the in-depth validation of molecular biology experiments and even the future clinical application of cancer therapy.

## Data Availability

The datasets presented in this study can be found in online repositories. The names of the repository/repositories and accession number(s) can be found in the article/Supplementary Material.
