# Supplementary figures and images for: Multi-omics analysis of LAMB3 as a potential immunological and biomarker in pan-cancer
Source: Front Mol Biosci. 2023 Jul 27;10:1157970. doi: 10.3389/fmolb.2023.1157970 (PMC10415034; doi:10.3389/fmolb.2023.1157970)

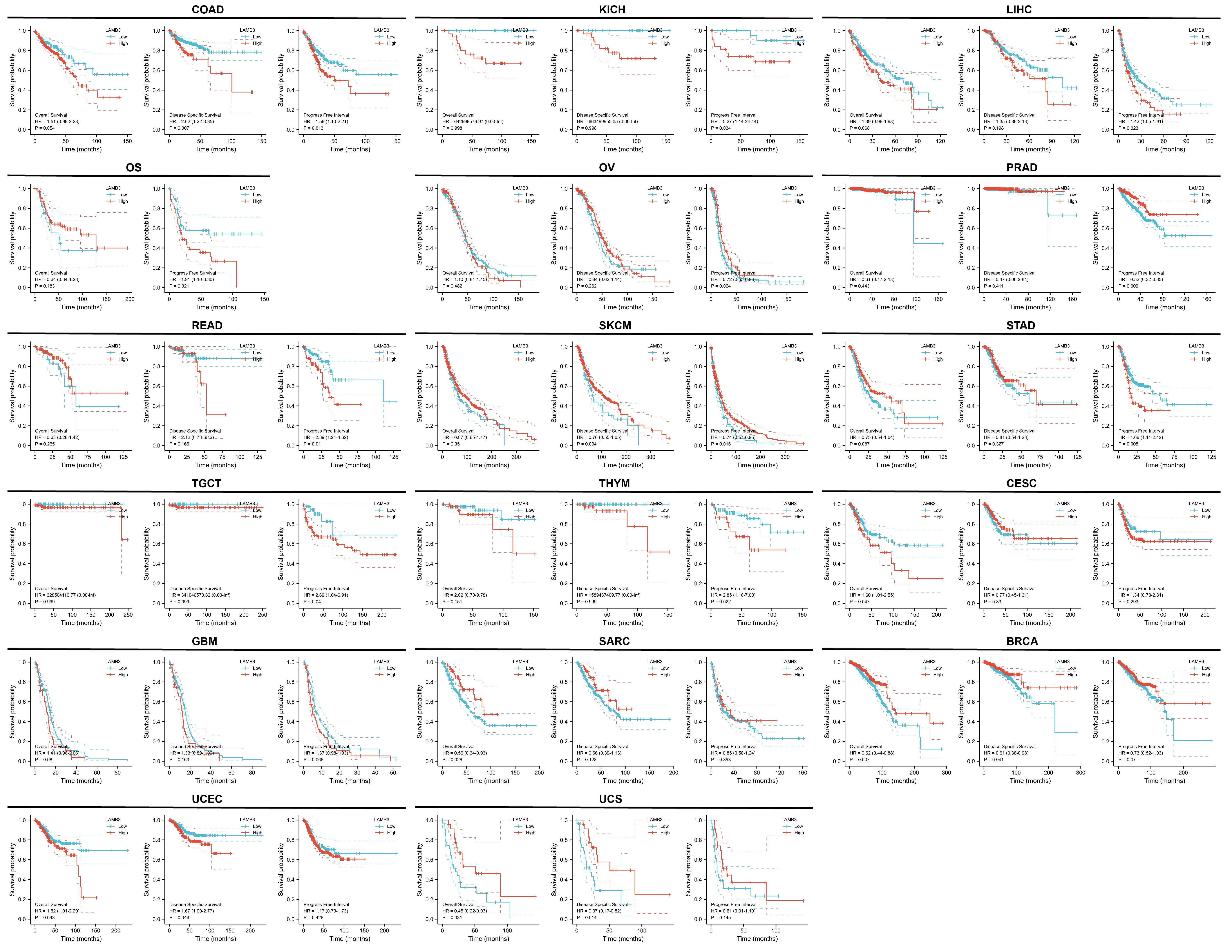

Supplement: Supplementary file 2 [file Image3.JPEG]

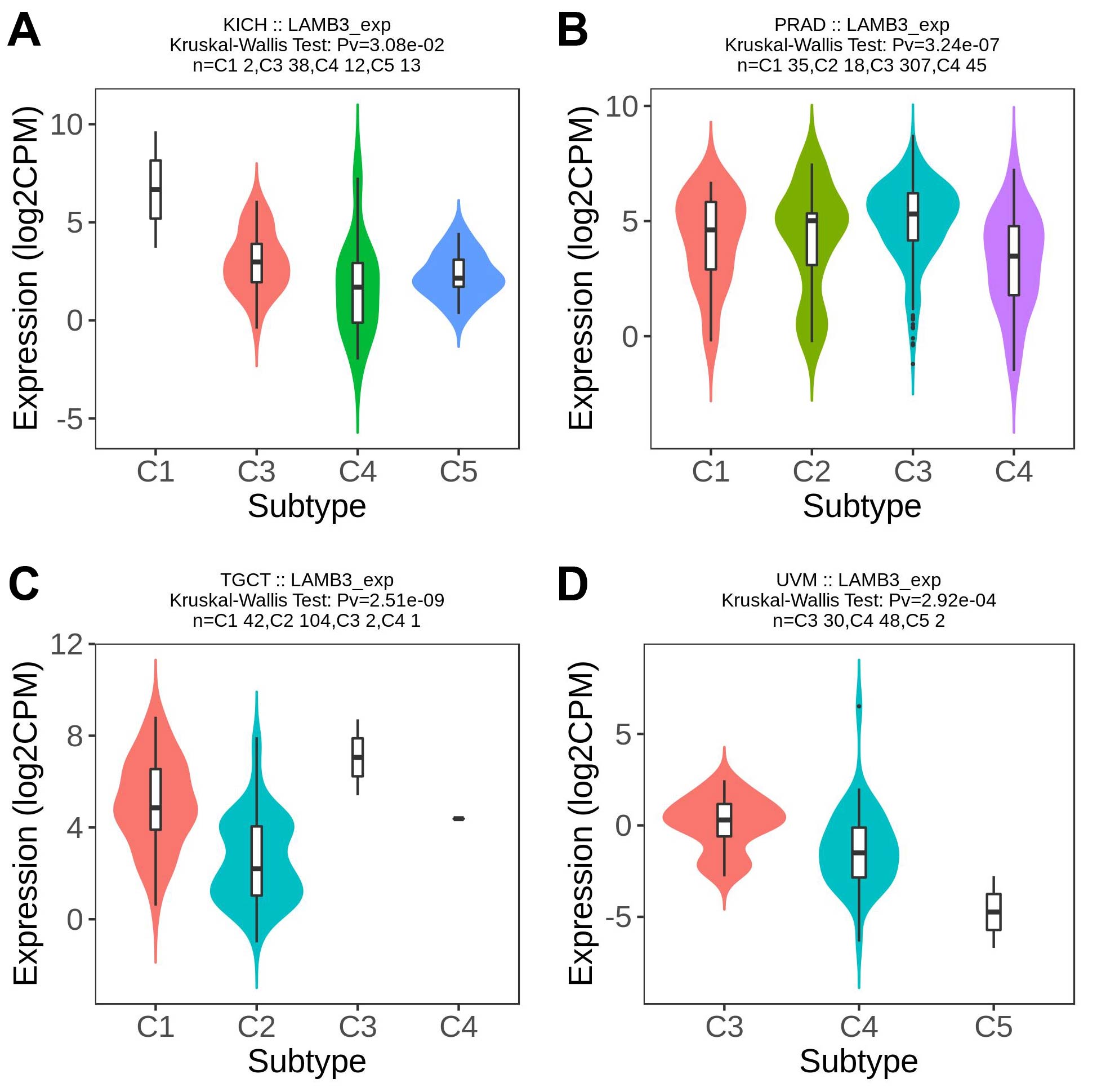

Supplement: Supplementary file 4 [file Image1.JPEG]

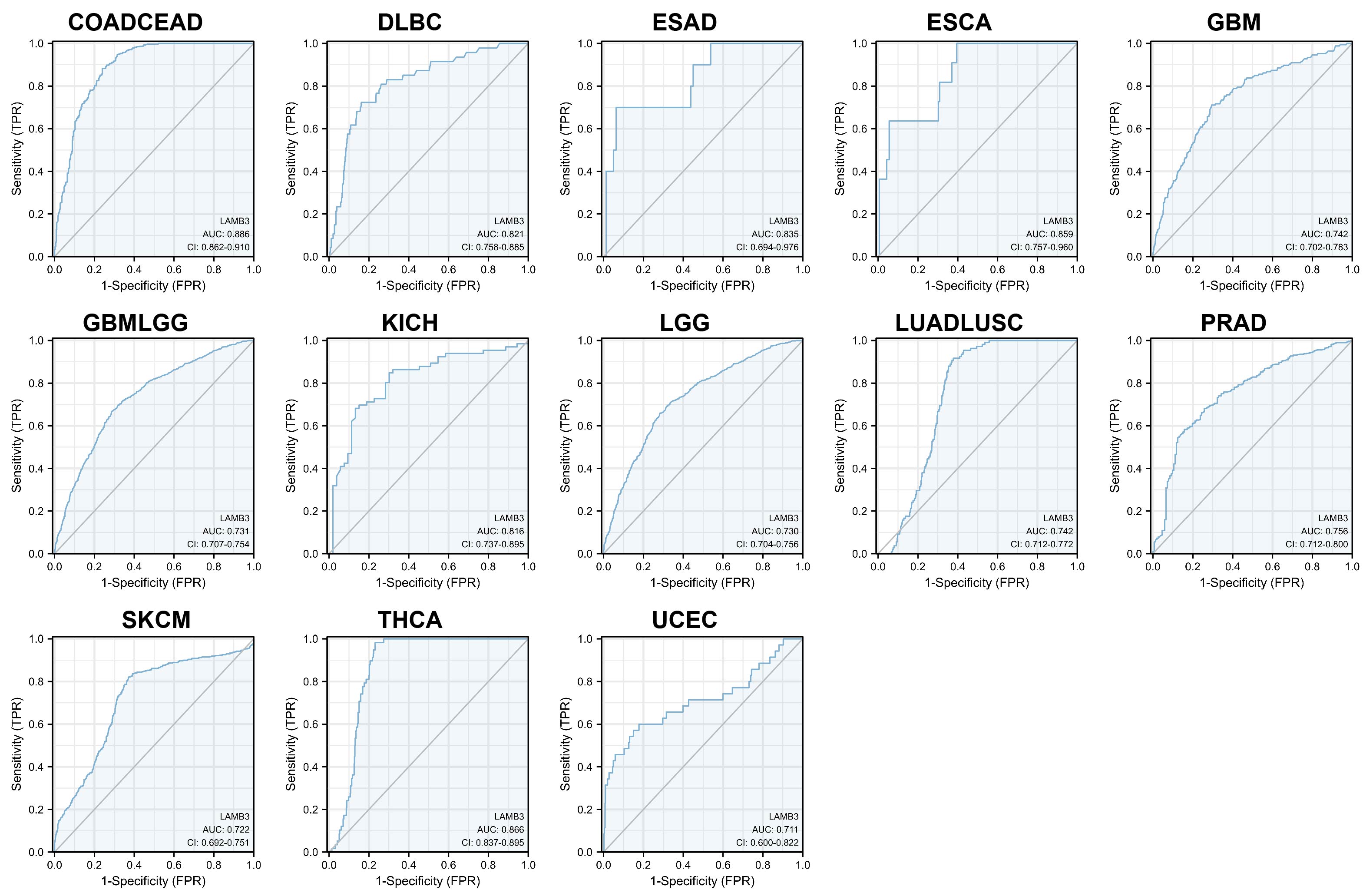

Supplement: Supplementary file 5 [file Image2.JPEG]
