# Supplementary material for: Multi-omics analysis of LAMB3 as a potential immunological and biomarker in pan-cancer
Source: Front Mol Biosci. 2023 Jul 27;10:1157970. doi: 10.3389/fmolb.2023.1157970 (PMC10415034; doi:10.3389/fmolb.2023.1157970)
Supplement: Supplementary file 7 [file Table3.DOCX]

| Characteristics | Total(N) | Univariate analysis | |  | Multivariate analysis | |
| --- | --- | --- | --- | --- | --- | --- |
|  |  | Hazard ratio (95% CI) | P value |  | Hazard ratio (95% CI) | P value |
| LUADLUSC | 1022 | 1.097 (1.022-1.178) | **0.011** |  | 1.097 (1.022-1.178) | **0.011** |
| COAD | 477 | 1.284 (1.032-1.597) | **0.025** |  | 1.284 (1.032-1.597) | **0.025** |
| COADREAD | 643 | 1.229 (0.998-1.513) | 0.053 |  | 1.229 (0.998-1.513) | 0.053 |
| HNSC | 501 | 1.128 (1.007-1.264) | **0.037** |  | 1.128 (1.007-1.264) | **0.037** |
| PAAD | 178 | 1.343 (1.157-1.560) | **<0.001** |  | 1.343 (1.157-1.560) | **<0.001** |
| BRCA | 1082 | 0.898 (0.816-0.989) | **0.029** |  | 0.898 (0.816-0.989) | **0.029** |
| SARC | 263 | 0.881 (0.781-0.994) | **0.039** |  | 0.881 (0.781-0.994) | **0.039** |
| THYM | 118 | 1.501 (0.992-2.272) | 0.055 |  | 1.501 (0.992-2.272) | 0.055 |
| UCS | 56 | 0.831 (0.691-1.000) | **0.050** |  | 0.831 (0.691-1.000) | **0.050** |
| KICH | 64 | 1.593 (0.971-2.615) | 0.066 |  | 1.593 (0.971-2.615) | 0.066 |
| OSCC | 328 | 1.125 (0.985-1.286) | 0.083 |  | 1.125 (0.985-1.286) | 0.083 |
| UVM | 80 | 1.403 (1.043-1.887) | **0.025** |  | 1.403 (1.043-1.887) | **0.025** |
| KIRC | 539 | 1.082 (0.994-1.177) | 0.068 |  | 1.082 (0.994-1.177) | 0.068 |
| KIRP | 288 | 1.200 (1.046-1.378) | **0.010** |  | 1.200 (1.046-1.378) | **0.010** |
| GBMLGG | 695 | 1.210 (1.049-1.395) | **0.009** |  | 1.210 (1.049-1.395) | **0.009** |
