# Supplementary material for: Multi-omics analysis of LAMB3 as a potential immunological and biomarker in pan-cancer
Source: Front Mol Biosci. 2023 Jul 27;10:1157970. doi: 10.3389/fmolb.2023.1157970 (PMC10415034; doi:10.3389/fmolb.2023.1157970)
Supplement: Supplementary file 9 [file Table4.DOCX]

| Characteristic | Low expression of LAMB3 | High expression of LAMB3 | p |
| --- | --- | --- | --- |
| n | 518 | 519 |  |
| T stage, n (%) |  |  | < 0.001 |
| T1 | 165 (16%) | 124 (12%) |  |
| T2 | 289 (27.9%) | 294 (28.4%) |  |
| T3 | 42 (4.1%) | 78 (7.5%) |  |
| T4 | 20 (1.9%) | 22 (2.1%) |  |
| N stage, n (%) |  |  | 0.253 |
| N0 | 343 (33.8%) | 325 (32%) |  |
| N1 | 99 (9.8%) | 127 (12.5%) |  |
| N2 | 56 (5.5%) | 58 (5.7%) |  |
| N3 | 4 (0.4%) | 3 (0.3%) |  |
| M stage, n (%) |  |  | 0.908 |
| M0 | 383 (47.6%) | 390 (48.4%) |  |
| M1 | 15 (1.9%) | 17 (2.1%) |  |
| Pathologic stage, n (%) |  |  | 0.010 |
| Stage I | 295 (28.8%) | 244 (23.8%) |  |
| Stage II | 122 (11.9%) | 163 (15.9%) |  |
| Stage III | 79 (7.7%) | 89 (8.7%) |  |
| Stage IV | 16 (1.6%) | 17 (1.7%) |  |
| Primary therapy outcome, n (%) |  |  | 0.925 |
| PD | 52 (6.4%) | 50 (6.2%) |  |
| SD | 25 (3.1%) | 29 (3.6%) |  |
| PR | 5 (0.6%) | 6 (0.7%) |  |
| CR | 323 (40%) | 317 (39.3%) |  |
| Gender, n (%) |  |  | < 0.001 |
| Female | 237 (22.9%) | 180 (17.4%) |  |
| Male | 281 (27.1%) | 339 (32.7%) |  |
| Race, n (%) |  |  | 0.620 |
| Asian | 10 (1.2%) | 6 (0.7%) |  |
| Black or African American | 44 (5.1%) | 41 (4.8%) |  |
| White | 381 (44.5%) | 375 (43.8%) |  |
| Age, n (%) |  |  | 0.012 |
| <=65 | 243 (24.1%) | 203 (20.1%) |  |
| >65 | 261 (25.9%) | 302 (29.9%) |  |
| Residual tumor, n (%) |  |  | 0.270 |
| R0 | 386 (49%) | 368 (46.8%) |  |
| R1 | 11 (1.4%) | 14 (1.8%) |  |
| R2 | 2 (0.3%) | 6 (0.8%) |  |
| Anatomic neoplasm subdivision, n (%) |  |  | 0.724 |
| Left | 209 (21%) | 210 (21.1%) |  |
| Right | 294 (29.6%) | 280 (28.2%) |  |
| Anatomic neoplasm subdivision2, n (%) |  |  | 0.456 |
| Central Lung | 96 (22.4%) | 113 (26.3%) |  |
| Peripheral Lung | 110 (25.6%) | 110 (25.6%) |  |
| number_pack_years_smoked, n (%) |  |  | 0.033 |
| <40 | 175 (22%) | 147 (18.5%) |  |
| >=40 | 219 (27.6%) | 253 (31.9%) |  |
| Smoker, n (%) |  |  | 0.804 |
| No | 48 (4.7%) | 45 (4.5%) |  |
| Yes | 456 (45.1%) | 462 (45.7%) |  |
| Age, median (IQR) | 66 (60, 73) | 68 (60, 73) | 0.022 |
